# Thymoquinone Enhances Tamoxifen Efficacy Against Triple‐Negative Breast Cancer by Targeting EMT Signaling

**DOI:** 10.1155/ijbc/1780945

**Published:** 2026-04-06

**Authors:** Mazharul Haque, Ritis K. Shyanti, Sudhanshu Sharma, Hongzhuan Wu, Manoj K. Mishra

**Affiliations:** ^1^ Cancer Research Center, Biological Sciences, Alabama State University, Montgomery, Alabama, USA, alasu.edu

**Keywords:** combinatorial therapy, docking and simulation, epithelial–mesenchymal transition, tamoxifen, thymoquinone, triple-negative breast cancer

## Abstract

The variety of available therapies for treating TNBC is constrained by the minimal or no expression of receptors such as estrogen (ER), progesterone (PR), and human epidermal growth factor 2 (HER2). However, estrogen receptor *β* (ER*β*) has emerged as a potential therapeutic target for TNBCs. Tamoxifen (TAM) is used as an endocrine therapy against ER‐positive cancer but has minimal impact on ER‐negative cancers like TNBC. Thymoquinone (TQ) is an active natural compound with anticancer properties, as evidenced by a cumulative number of reports. A combinatorial approach to targeting EMT in TNBC is one strategy that may improve TAM activity. In this study, we evaluated the binding affinity and stability of TQ and TAM with ER*β* in silico and assessed their cytotoxic potential individually and in combination in vitro. Docking and molecular dynamics (MD) studies confirmed interactions between ER*β* and TQ, TAM, and GEN. The IC_50_ values for TQ and TAM were approximately 18.44 and 11.76 *μ*M, respectively, for MDA‐MB‐468, and 22.06 and 15.73 *μ*M, respectively, for MDA‐MB‐231. An in vitro study demonstrated enhanced cytotoxic effects when TQ (20 *μ*M) and TAM (13 *μ*M) were combined. Further analysis revealed downregulation of the mesenchymal markers CDH2 (N‐cadherin) and vinculin, which are responsible for cell migration. Additionally, low vimentin and enhanced E‐cadherin expression lead to downregulation of core EMT regulators, including SNAIL1 and ZEB1. In conclusion, TQ showed potential chemomodulatory effects on TAM against TNBC by reducing the expression of EMT‐associated markers.

## 1. Introduction

Tamoxifen (TAM) has been used as an endocrine therapy against estrogen receptor (ER)‐positive breast cancer [1]. However, low efficacy against ER‐negative cancer cells poses unmet challenges [2]. Additionally, toxicity and resistance against chemotherapeutic drugs are the primary concerns, reducing the efficiency of TAM therapy [3]. Combining phytochemicals is one of the current strategies to enhance the efficacy of TAM in treating breast cancer patients [4]. Thymoquinone (TQ) is a principal bioactive compound derived from Nigella sativa, commonly known as black cumin [5]. It has attracted considerable interest in combination therapy due to its antioxidant, antiallergic, antidiabetic, and anticancer properties [6–12]. TQ has been shown to possess potent anticancer properties, both individually and in combination with anticancer drugs such as cisplatin [13, 14]. More importantly, combining TQ with anticancer drugs has reduced their side effects in vivo [15]. In a study, new targets of TQ in triple‐negative breast cancer (TNBC) were identified through genome‐wide methylation analysis. From the analysis of MBD1 ChIP sequencing, it was revealed that expression of interleukin‐17 receptor type D (IL17RD) is epigenetically upregulated by TQ, resulting in reduced TNBC cell growth and metastasis [16]. In another study, improved antitumor effects through PI3K/Akt1 pathway inhibition in response to TQ treatment were observed in metastatic breast cancer cells with PIK3CA mutations at p. H1047R and p. H1047L [17]. TNBC is considered as one of the aggressive molecular subtypes of breast cancer characterized by the absence or minimal expression of ER, progesterone receptor (PR), and human epidermal growth factor 2 receptor (HER2), which limits targeted therapeutic options [18, 19]. Estrogen receptor *β* (ER*β*) has emerged as a potential therapeutic target against TNBC [20, 21]. Numerous studies have reported the presence of ER*β* protein in several human breast cancers, as validated through immunohistochemistry [22]. Recent population‐based studies on primary breast cancers have shown that ER*β* is expressed in over half of TNBCs [23]. The expression is highest in the basal subtype of TNBCs, and patients with tumors expressing ER*β* have better overall survival, suggesting that ER*β* may be a potential target for endocrine therapy in TNBC [24]. Previous studies demonstrated that ER*β* inhibits the growth and migration of TNBC in vitro [21]. TNBC accounts for 9–20% of breast cancer cases and is more common in African–American and Hispanic populations compared with non‐Hispanic white women [25, 26]. The extracellular matrix (ECM) undergoes significant structural changes during cancer progression [27]. TNBC can acquire mesenchymal properties in addition to the interaction of ECM and cancer cells [28]. Altered ECM triggers epithelial‐to‐mesenchymal transition (EMT) and facilitates migration, invasion, and resistance to chemotherapy [29, 30]. EMT is a biological phenomenon characterized by the loss of adhesion properties, specifically the downregulation of E‐cadherin and adherent junctions and the gain of mesenchymal characteristics, such as the upregulation of N‐cadherin, vinculin, and vimentin [31, 32]. These changes enable epithelial cells to detach from their original location and acquire increased mobility [33]. EMT is regulated by the TGF‐*β*, Notch, and Wnt signaling pathways, which activate the Snail, Slug, ZEB, and Twist transcriptional factors [34, 35]. The inhibition of EMT in the combined treatment of TQ and TAM provides a potential strategy for targeting TNBC. This study investigated the synergistic effect of TQ and TAM in inhibiting the EMT pathway as a potential strategy for treating TNBC.

## 2. Materials and Methods

### 2.1. Chemicals and Reagents

Dulbecco′s modified Eagle′s medium (DMEM, Cat# 11995‐065), fetal bovine serum (FBS, Cat# 10082‐147), and penicillin–streptomycin (pen‐strep, Cat# 15140) were procured from Gibco. TQ (Cat# 305070050, purity 99%), TAM (Cat# J6350903, purity 99%), and MTT (Cat# M2003) were purchased from Sigma. Viability/Cytotoxicity Assay Kit (Cat# 30002) was purchased from Biotium. RIPA lysis buffer (Cat# 89901) was purchased from Thermo Scientific. Dimethyl sulfoxide (DMSO, Cat# 317275) was purchased from Millipore Sigma. Phosphate‐buffered saline (PBS, Cat# 1860454) was purchased from MP Biomedicals. iScript cDNA Synthesis kit (Cat# 1725037) and iTaq Universal SYBR Green Supermax (Cat# 1725124) were purchased from Bio‐Rad. Vimentin pAb (Cat# PA5‐81968) and GAPDH polyclonal antibody were procured from Invitrogen. E‐cadherin Rabbit pAb (Cat# NBP2‐16258) was purchased from Novus Biologicals. Vinculin polyclonal antibody (Cat# PA5‐29688) obtained from Thermo Scientific and antirabbit HRP‐linked secondary antibody (Cat# 7074P2) were purchased from Cell Signaling Technology.

### 2.2. Protein and Ligand Structure Preparation and Binding Pocket Analysis

The human 3D crystal structure of ER*β* was obtained from the RSCB database using the PDB ID: 3OLS [36]. This structure represents the ligand‐binding domain of human Er*β*. The structure was checked, and we used Chain B for our studies, as it contained bound small ligands and heteroatoms among the seven chains. The missing residues in Chain B were completed by performing the homology modeling using MODELLER 9v15 [37]. To identify potential binding pockets on the surface of ER*β*, we used the CASTp tool [38]. This helped us predict potential binding sites for TQ, TAM, and the reference ligand genistein (GEN) on ER*β*. The ligand structures were cleaned of water residues, and hydrogen atoms were added using the Avogadro2 tool [39]. We used GPU‐accelerated AUTODOCK Vina for molecular docking [40]. All the SDF files were converted to PDBQT format as accepted by Vina. The docking output logs and PDBQT files were visualized using BIOVIDIA Discovery Studio [41].

### 2.3. Molecular Dynamics (MD) Simulations

We used the GROMACS 2023.1 package (https://www.gromacs.org/) and the CGenFF tool to convert the ligand text files.GRO, and STR format [42]. CHARMM36 was the force field set we used to perform the MD simulations [43]. A 100‐ns run was conducted to evaluate the structural dynamics and stability of the ligands in complex with ER*β*. Energy minimization was performed at 10 kJ/mol/nm using the long descent steep algorithm for 10,000 conjugate gradient steps to avoid steric hindrance. System equilibration for pressure and temperature (NPT and NVT) was performed at 1 bar and 310 Kelvin over a 100 ns time scale. [44]. Postsimulation analysis was performed to calculate the root mean square deviation (RMSD), root mean square fluctuations (RMSF), radius of gyration (Rg), number of H‐bonds, and the interaction energy in Coulombs and Lennard‐Jones units for short ranges. The results were analyzed, and all the plots were plotted using the Xmgrace tool [45].

### 2.4. Cell Culture and Treatment

Human TNBC cell lines HTB‐132 (MDA‐MB‐468; RRID: CVCL_0419) and HTB‐26 (MDA‐MB‐231; RRID: CVCL_0062) were procured from American Type Culture Collection (ATCC), United States (procurement date: June 30, 2023). The cell lines originated from breast tissues from a female patient with metastatic breast carcinoma. These cells were cultured in DMEM supplemented with 10% FBS and 1% pen–strep and maintained at 37°C in a humidified incubator with 5% CO_2_. Before setting up the experiment, these cell lines were checked for mycoplasma contamination using the APExBIO PCR Mycoplasma Detection Kit (Catalog No: K2821). TQ and TAM compounds were separately dissolved in dimethyl sulfoxide (DMSO) at a final concentration of 0.1% (v/v) for subsequent use.

### 2.5. Cell Proliferation Assay to Determine the Optimal Dose for TQ and TAM Against TNBC Cells

Approximately 10^4^ cells were grown in 96‐well culture plates and incubated for 24 h in a CO_2_ incubator at 37°C in a humidified incubator maintained at 5% CO_2_. The cells were then treated with different concentrations of TQ and TAM (5, 10, 20, 40, and 80 *μ*M) in three biological replicates, each with triplicate samples, and cell viability was assessed using the MTT assay after 24 h of treatment. Briefly, 20 *μ*L of MTT solution (5 mg/mL in PBS) was added to each well, and the plate was incubated for 4 h. The culture medium containing MTT was discarded, and 100 *μ*L DMSO was added to dissolve the Formazan crystals at room temperature for 30 min. The optical density (OD) was measured at 570 nm using a Synergy multimode plate reader (Agilent BioTek, United States). IC_50_ for both drug treatments on both cell lines (MDA‐MB‐468/231) was estimated by plotting % cytotoxicity (*y*‐axis) versus drug concentration (*x*‐axis) , as presented in Figure S1a,b. The linear regression was performed to obtain the best‐fit linear equation (Y = mX + c), where *m* is the slope, and *c* is the intercept. Then, the value for *Y* was set to 50 (for 50% inhibition) to estimate *X* by using the equation (X = 50 − c/m) [46].

### 2.6. Cell Morphological Analysis

Approximately 5 × 10^4^ cells/well were seeded in 12‐well culture plates and incubated for 24 h before treatment. The cells were seeded in triplicate. Further cells were treated with TQ and TAM alone or in combination for 24 h. The cells were then washed twice with PBS and fixed with 4% paraformaldehyde (PFA). After 8 min of fixing, the cells were washed with PBS to remove PFA. Then, as recommended, two drops of F‐Actin (AlexaFluor 488 phalloidin, cat# R37110, Invitrogen) were added to it and incubated for 30 min in the dark. Again, the cells were counterstained with DAPI (1 *μ*g/mL) and incubated in the dark for 15 min. Then, the stain was removed by washing the cells three times with PBS and imaging them using a fluorescence microscope (EVOS FL, Invitrogen).

### 2.7. Live/Dead Assay

Approximately 10^5^ cells were seeded in each well of 12‐well plates and treated with TQ (20 *μ*M) and TAM (13 *μ*M) alone and in combination for 24 h. The experiment was performed in duplicates. Cells were washed twice with serum‐free DMEM. The staining solution, consisting of 2 *μ*M calcein acetoxymethyl ester (calcein AM) and 4 *μ*M ethidium homodimer III (EthD‐III), was prepared by adding 5 *μ*L of 4 mM Calcein AM and 20 *μ*L of 2 mM EthD‐III to 10 mL of PBS, and then vortexing to ensure proper mixing. A sufficient staining solution was added to cover the cell monolayer. The cells were incubated for 30–45 min at room temperature. The staining solution was removed, and the sample was washed with PBS. Images of the cells were taken by a fluorescence microscope (EVOS FL, Invitrogen) [47].

### 2.8. Apoptosis Assay

After treatment with TQ, TAM, or the combination, the mechanism of cell death was elucidated using an apoptosis assay by flow cytometry. The early and late apoptotic as well as necrotic cell populations were identified using Annexin V‐FITC (BioLegend, Cat# 640945) and propidium iodide (PI, Thermo Scientific, Cat# J66764MC). After 24 h, cells from each treatment group were harvested and washed twice with PBS. They were then incubated in the dark with a 0.2‐m cocktail of annexin/PI solution for 30 min, following the manufacturer′s directions. Stained cells were analyzed using an ACEA Novocyte flow cytometer (ACEA Biosciences Inc., San Diego, California, United States), and fluorescent signals for FITC and PI were acquired. Twenty thousand events were acquired for each sample, and FITC/PI‐positive cells were analyzed using ACEA NovoExpress software (ACEA Biosciences Inc., San Diego, California, United States). The study was performed in three biological replicates in triplicate.

### 2.9. Wound Healing Assay

Cells were plated in 12‐well culture plates to assess cell motility and grown till adequate confluency. The culture medium was removed, and the cells were washed with PBS. Then, fresh serum‐free medium was added, and the mixture was incubated in a CO_2_ incubator at 37°C with 5% CO_2_ for 4 h. Then, a scratch was created in the cell layer using a sterile tip. The cells were then treated with TQ (20 *μ*M), TAM (13 *μ*M), and TQ + TAM (20 and 13 *μ*M), and images were captured at different time points (0, 12, 24, and 48 h) to assess wound closure. The wound‐closure area was then measured using ImageJ. The percentage of the wound area was compared with the initial wound at 0 h. The cells were seeded in triplicate. The pictures shown are representative of experiments performed in triplicate.

### 2.10. Cell Adhesion Analysis

Before cell seeding, the E‐plate was exposed to UV for 15 min in a biosafety hood. Then, each well was coated with collagen I (4 *μ*g/mL), incubated for 1 h in a CO_2_ incubator, and washed twice with 150 *μ*L sterile PBS. After that, we added 50 *μ*L prewarmed DMEM to each well and incubated for 15 min in an incubator to achieve equilibrium before starting the experiment. Approximately 10,000 cells/well were seeded in two biological replicates, each in triplicate, and treated individually and in combination. The plates were placed in the xCELLigence RTCA instrument to measure real‐time cell adhesion kinetics at 15‐min intervals to assess cell adhesion properties [32].

### 2.11. Gene Expression Analysis

The expression profiles of significant EMT genes in TNBC were demonstrated in MDA‐MB‐468 and MDA‐MB‐231 cells. In brief, cells (at a density of approximately 10^5^ cells) were cultured overnight in a CO_2_ incubator at 37°C in 6‐well culture plates in triplicate, followed by treatment with TQ (20 *μ*M), TAM (13 *μ*M), and the combination for 24 h. Cells were then collected from each well using a scraper, pelleted, and washed twice with PBS. Total RNA was isolated using TRIzol reagent according to the manufacturer′s directions. For phase separation, 0.2 mL of chloroform was added, vortexed, and centrifuged for 15 min. at 14,000 rpm at 4°C after incubating at room temperature for 5 min. The aqueous phase was collected, and 0.5 mL of isopropyl alcohol was added to the RNA pellet, followed by a 70% ethanol wash. The RNA pellets were then eluted in 20 *μ*L of nuclease‐free water and stored at −80°C for further use. The RNA was quantified with a NanoDrop spectrophotometer (NanoDrop One; Thermo Fisher Scientific). The iScript cDNA synthesis kit was used to synthesize cDNA. Synthesized cDNA was used to quantify gene expression using specific primers for target genes by quantitative real‐time PCR (q‐PCR; CFX96, Bio‐Rad). The list of primers used in this study is provided in Table S1. The specificity was evaluated by analyzing the PCR product melt curves. The standard curve (fold change) was estimated using the *ΔΔ*Ct method. GAPDH was used as a loading control, and the Ct value of it was subtracted from each treated group to estimate *Δ*Ct. Then, *ΔΔ*Ct values were calculated by subtracting the *Δ*Ct of the control sample from the *Δ*Ct of the treated sample. Finally, relative gene expression was calculated using the 2 ^−*Δ* *Δ*Ct^ formula to determine fold change.

### 2.12. Western Blot Analysis

Approximately 5 × 10^5^ cells were seeded into each well of six‐well culture plates and incubated for 24 h. Then, cells were collected and washed twice with PBS after 24 h of treatment with TQ, TAM, or both. The cells were then lysed in precold RIPA buffer in a protease and phosphatase inhibitor cocktail (Cat# 78410, Thermo Scientific). Cells were centrifuged at 14,000 × g for 15 min at 4°C to collect the extracted total protein in the supernatant. After protein estimation, an equal amount of total protein (20 *μ*g) was separated onto 10% SDS‐PAGE and transferred to the PVDF membrane (cat# 1620177, Bio‐Rad). After transfer completion, the membranes were blocked in 5% non‐fat skim milk and incubated overnight with the primary antibodies (in TBST/milk) at 4°C. The vimentin antibody (1:2000, equivalent to 0.1 *μ*g/mL) was used. The vinculin antibody (1:2000) concentration, equivalent to 0.165 *μ*g/mL, was used. However, the E‐cadherin antibody was diluted 1:1000 (0.7 *μ*g/mL). After incubation, the membranes were washed three times with TBST for 5 min. Membranes were then incubated with HRP‐conjugated secondary antibody (1:3000) equivalent to 0.66 *μ*g/mL for 1 h at room temperature. Again, the membranes were washed three times with TBST after incubation. The ECL substrate was applied to the membrane according to the manufacturer′s instructions to detect the HRP‐labeled secondary using a chemiluminescent imaging system (Bio‐Rad, United States). GAPDH was used as a loading control.

### 2.13. Statistical Analysis

Data acquired in this study were analyzed using GraphPad Prism 9.3.1. (GraphPad Software, Inc., San Diego, California, United States). The mean ± S.E.M. of at least three separate experiments is shown. As shown in the legends, a one‐way analysis of variance (ANOVA) followed by Tukey′s post hoc test was used to establish the significance of the difference. A *p* value of < 0.05 was considered significant (as indicated in the figures and legends).

## 3. Results

### 3.1. Docking Analysis to Calculate the Binding Affinities Between ER*β* and TQ, and TAM, and GEN

Molecular docking was performed using AUTODOCK Vina with GPU acceleration to calculate the binding affinities of TQ and TAM against ER*β*. Docking parameters, such as the box coordinates, center, and size, are used with the Python libraries (Biopython and RDKit). The calculated coordinates for docking analysis were the center (*x* = 4.16, *y* = −25.54, *z* = 5.57) and the box size (*x* = 44.54, *y* = 54.32, *z* = 51.55). The crystal structure of ER*β* was retrieved from the RCSB PDB with **PDB ID: 3OLS**. The heterodimer structure was cleaned of any attached ligand, and Chain B was finally selected after checking for missing amino acid residues. TQ showed a more favorable binding with ER*β*, yielding a binding energy of −9.2 kcal/mol and an RMSD of 1.44 Å compared with TAM. The interacting residues were Leu343, Phe356, Leu339, Met336, Ala302, Leu476, and Leu298. TAM showed a binding affinity of −8.7 kcal/mol with an RMSD of 1.54 Å, and the interacting residues were Lys300, Met296, Ile282, Asp303, Val280, Lys304, and Pro486. On the other hand, GEN showed a binding affinity of –8.5 kcal/mol, and the interacting residues were Gly212, Ala42, Leu38, and His215. This showed that TAM and TQ exhibited favorable binding energies and interactions compared to GEN on the surface of ER*β*. All these observations are shown in Figures 1a, 1b, 1c, 1d, 1e, and 1f.

Figure 1Representation of the 2D and 3D structures of the TQ, TAM, and reference ligand GEN docked complexes with ER*β*. (a, b) Visualizes the docking analysis of TQ with a binding score of −9.2 kcal/mol. The key interactions observed were Leu343, Phe356, Leu339, Met336, Ala302, Leu476, and Leu298. (c, d) Show the binding interactions of TAM with the ER*β* in 3D and 2D plots. The calculated binding energy after docking analysis was −8.7 kcal/mol, and key residues that showed interactions were Lys300, Met296, Ile282, Asp303, Val280, Lys304, and Pro486. (e, f) GEN in 3D and 2D, with a binding energy of –8.5 kcal/mol, forms hydrogen bonds with residues Gly212, Ala42, Leu38, and His215 in the ER*β* ligand‐binding domain.

**Figure:**
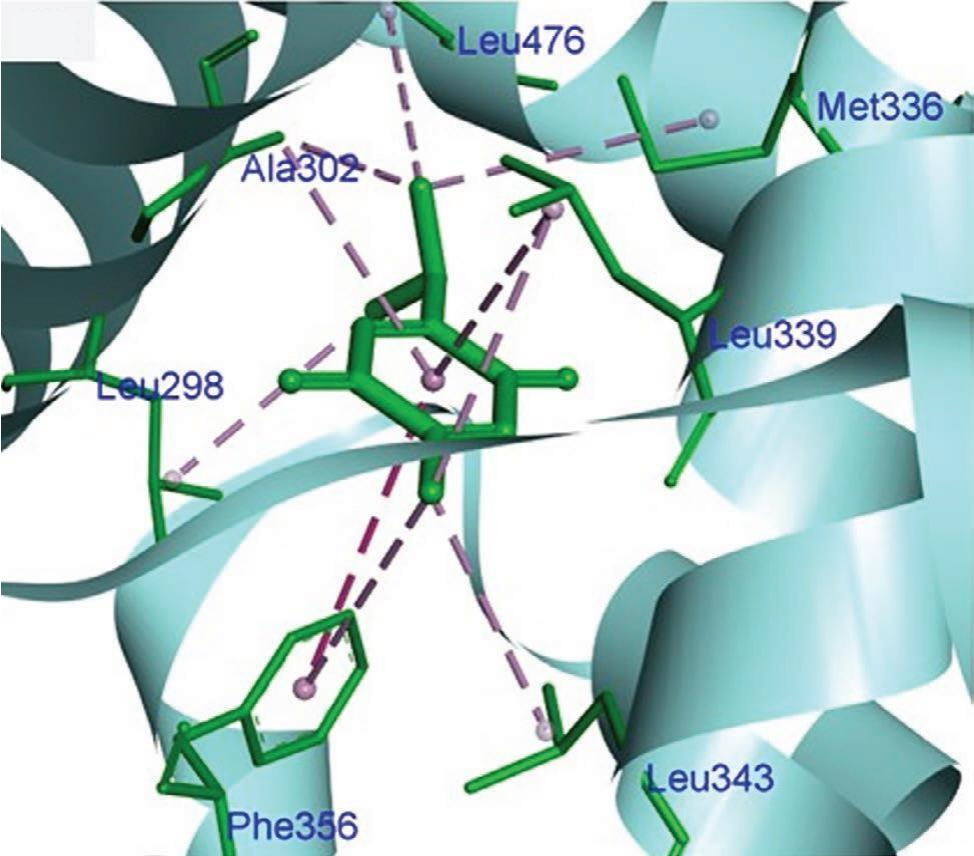
(a)

**Figure:**
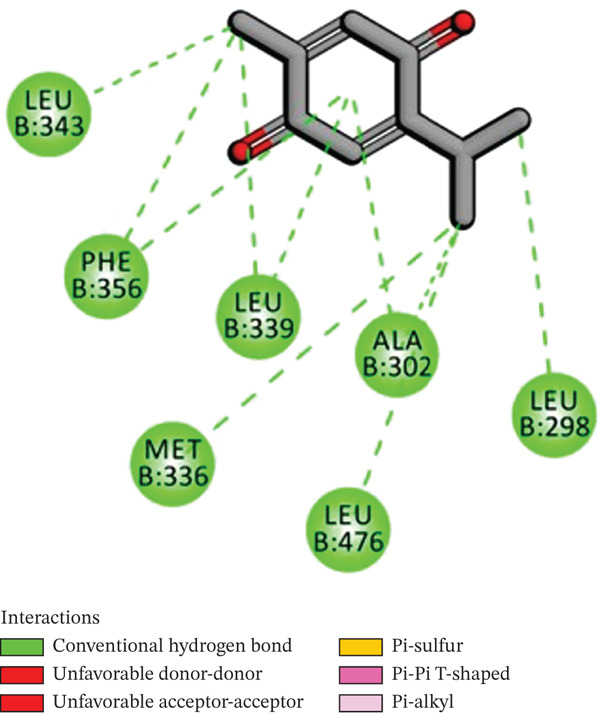
(b)

**Figure:**
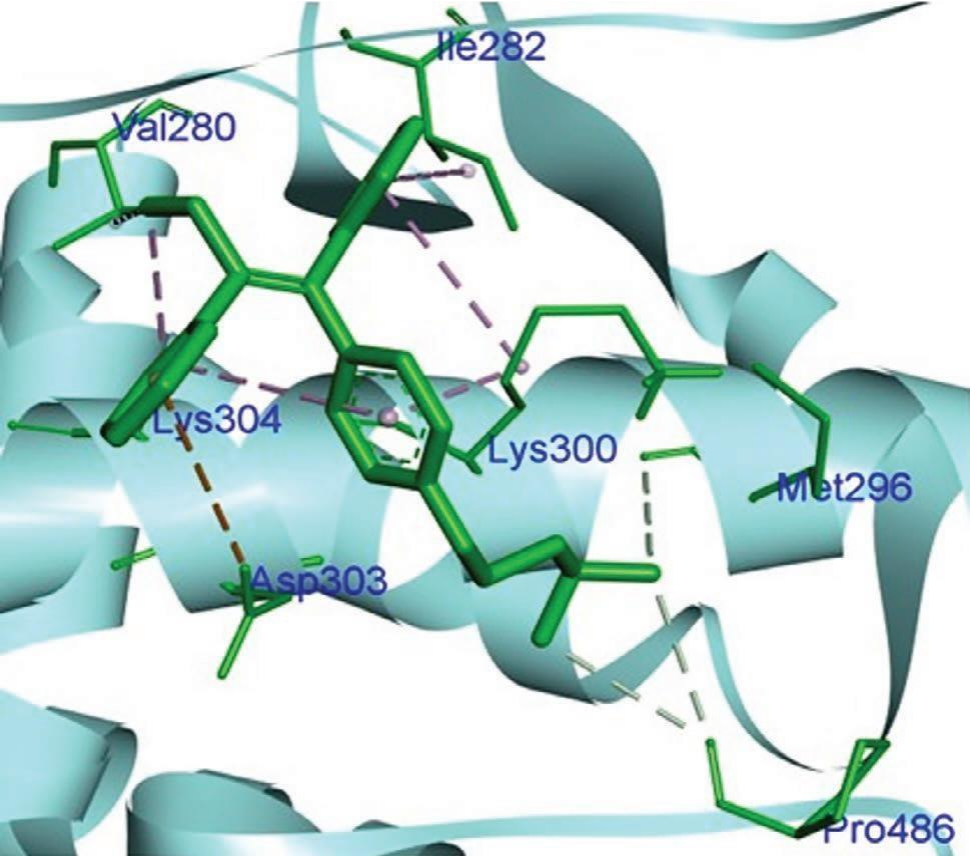
(c)

**Figure:**
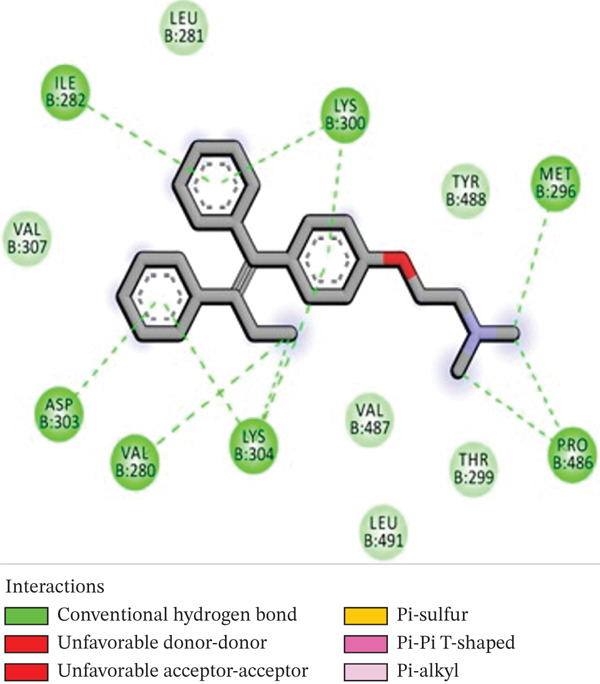
(d)

**Figure:**
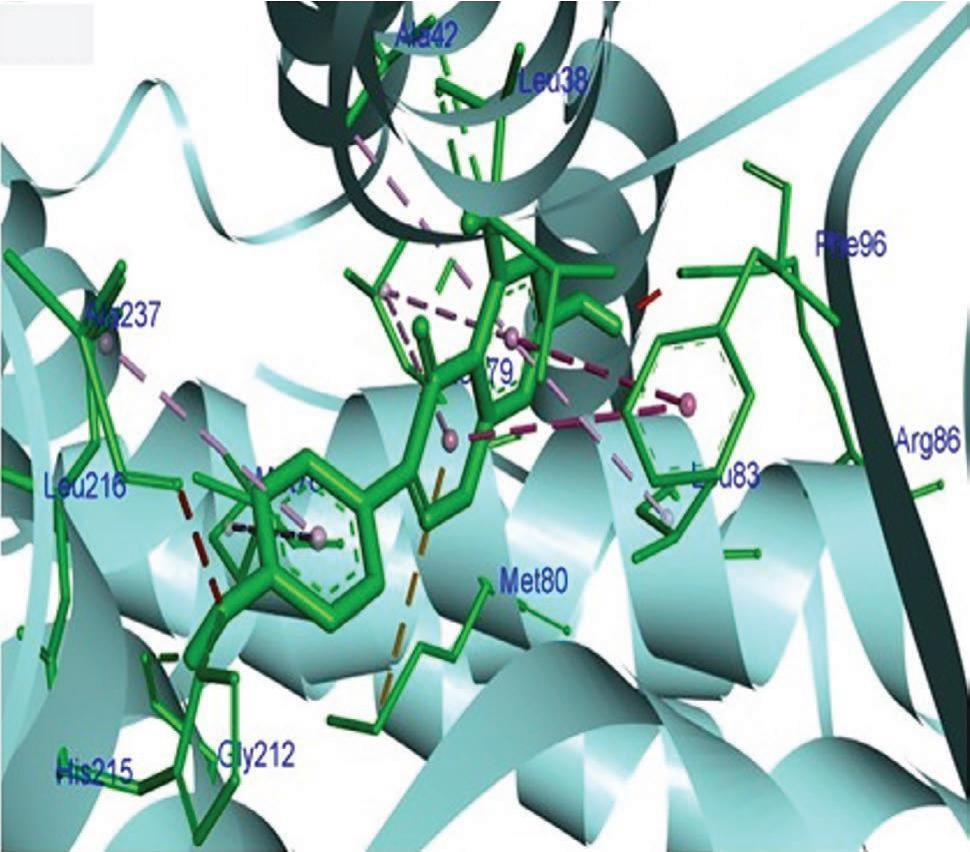
(e)

**Figure:**
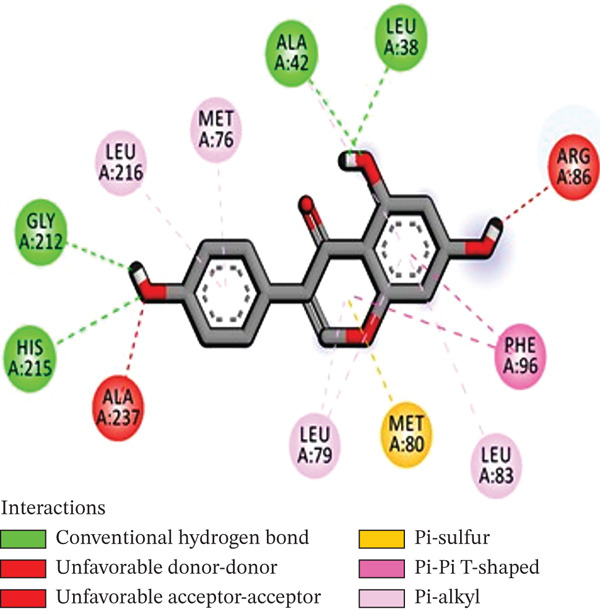
(f)

### 3.2. MD Simulations Analysis of ER*β* and Ligand Complexes

The structural dynamics, integrity, and stability of the ER*β* when complexed with TQ, TAM, and GEN were investigated using MD simulations for 100 ns. Key parameters were obtained, including the RMSD, RMSF, Rg, number of hydrogen bonds, minimum distance among residues, and interaction energies. The RMSDs were measured for Complexes I (TQ with ER*β*), II (TAM with ER*β*), and III (GEN with ER*β*). The backbone alone displayed higher RMSD values, ranging from 0.25 to 0.4 nm. The RMSD of Complex I was in the range from 0.03 to 0.08 nm. This demonstrated larger structural intactness than ER*β* alone. On the other hand, Complex II displayed an RMSD of 0.05–0.15 nm, showing better stability than ER*β* alone but less than Complex I. The RMSF reflects numerical calculations like RMSD but measures the individual‐residue flexibility. Notably, the GEN‐bound Complex III exhibited higher RMSD values (0.1–0.45 nm), comparable with both Complexes I and II. There are severe fluctuations during the stability in the case of Complex III, suggesting a less stable binding than in Complexes I and II. All three complexes showed minimal per‐residue fluctuations (< 0.2 nm) across most of the sequence. Complex II has marginally higher fluctuations in this loop region than Complexes I and III. Overall, the difference was comparatively marginal. Complexes I and III maintained lower RMSF in that region than Complex II, implying marginally more rigid binding at those residues. When compared, all the complexes maintained Rg in a narrow range of 1.80–1.95 nm, thereby showing better structural integrity. Complex I exhibited a slightly higher Rg value, indicating a marginal loss of structural potential due to conformational changes during binding. Complex II showed moderate compactness compared with unbound ER*β*. These calculations are shown in Figures 2a, 2b, and 2c.

Figure 2MD simulation analyses of ER*β* alone versus ER*β* bound to TQ, TAM, and GEN. (a) RMSD of the ER*β* backbone over 100 ns, the unliganded ER*β* (green) shows higher RMSD (0.25–0.40 nm) due to flexibility, whereas ER*β*–TQ (blue) stays around 0.03–0.10 nm and ER*β*–TAM (orange) around 0.05–0.20 nm. The GEN complex (red) showed higher RMSD (0.04–0.10 nm), indicating enhanced stability for all ligand‐bound forms (TQ and GEN bound complexes exhibit the lowest deviations; (b) RMSF per residue of ER*β* with TQ (blue), TAM (orange), and GEN (red) bound fluctuated less than 0.2 nm. A slightly higher peak around residues 160–165 is observed in all complexes, with TAM showing a marginally higher peak than TQ or GEN. Ligand binding reduces fluctuations, with the TQ and GEN complexes showing slightly lower RMSFs than TAM. (c) Radius of gyration (Rg) of ER*β*: the apo receptor (green) becomes somewhat more compact over time (~1.91 → 1.85 nm), whereas ER*β* with TQ (blue) or GEN (red) remains consistently compact (~1.86–1.95 nm). The TAM‐bound ER*β* (orange) follows a similar trend, indicating that none of the ligands disrupts the global protein folding; all complexes retain a tight, stable conformation.

**Figure:**
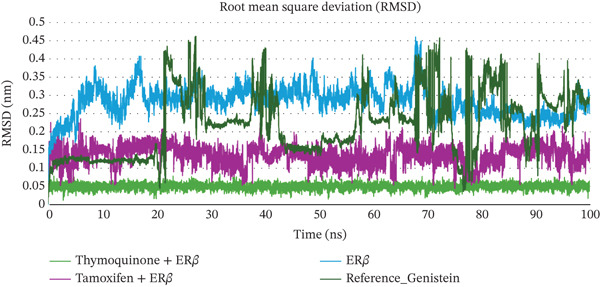
(a)

**Figure:**
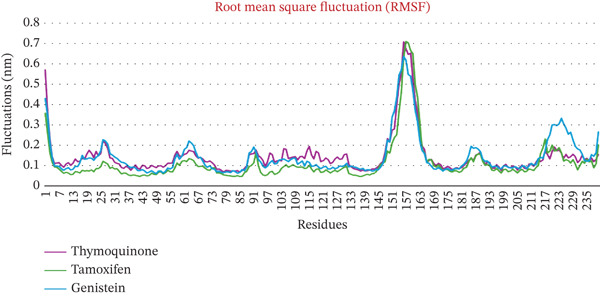
(b)

**Figure:**
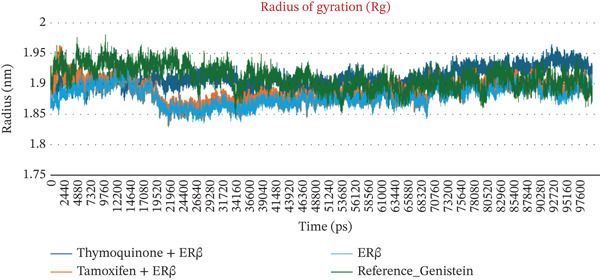
(c)

The number of H‐bonds during complex formation between the receptor and the ligand plays a crucial role. These indicates a threshold distance between the acceptor and donor atoms that predicts the overall stability and interactions between the molecules. Initially, they ranged from 0.5 to 2 bonds, for 30 ns in both complexes However, as the simulation progressed, the number increased sharply, reaching 2‐4 bonds. In the case of Complex I, the bond number ranged from 0.5 to 4, whereas in Complex II, the number of bonds was 1–4, slightly higher than in Complex I. The minimum distance between residues tell us the shortest atomic distance between the ligand and the receptor. This shows potential binding consistency and proximity. TQ complexed with ER*β* showed a periodic distance spike in the range of ~2.5 Å, reflecting the dynamic movement of the ligand but without complete detachment from the receptor. TAM maintained a constant minimum distance of 0.1–0.5 Å, indicating better binding than Complex I. When discussing Coulomb′s interaction energies that show hydrophobic and van der Waals interactions, Complex I displayed binding energies in the −67,000 to −68,000‐KJ/mol range. In contrast, the value ranged from −710,000 to −720,000 kJ/mol for Complex II. Complexes I and II maintained a steady LJ‐SR of ~50,000–60,000 KJ/mol, suggesting stronger electrostatic binding for ER*β*. All these individual parameters are shown in Figures 3a, 3b, 3c, and 3d.

Figure 3(a–d) Dynamic interaction parameters for ER*β*–ligand complexes (Complex I: TQ–ER*β*; Complex II: TAM–ER*β*; and Complex III: GEN–ER*β*). (a) Number of hydrogen bonds between each ligand and ER*β* over 100 ns. TQ (blue) generally formed fewer H‐bonds than TAM (orange), whereas GEN (red) was intermediate. All complexes ranged between 1 and 4 H‐bonds, with TAM consistently at the upper end of that range and TQ often at the lower end. (b) Minimum ligand‐receptor distance as a function of time. The TQ complex (blue) showed occasional spikes up to 2.5 Å (transient ligand movements), whereas TAM (orange) and GEN (red) remain very close to ER*β* (distance < 1 Å almost consistently), indicating tighter, consistent binding for TAM and GEN. (c) Coulomb′s energy was more negative, which revealed stronger electrostatic interactions. In Complex I, the energy was in the range −4.7 × 10^5^ to −4.8 × 10^5^ kJ/mol. In the case of Complex III, the energy was −5.0 × 10^5^ kJ/mol, whereas in Complex II, the values were in the range −7.1 × 10^5^ to −7.3 × 10^5^ kJ/mol. (d) Lennard‐Jones (van der Waals) interaction energies. All three complexes show favorable van der Waals stabilization on the order of 5 × 10^4^ kJ/mol. TQ (blue) has LJ energies in the 5.5–6.0 × 10^4^ kJ/mol range, TAM (orange) in 5.0–5.6 × 10^4^, and GEN (red) in between. The slightly lower LJ energy for Complex II indicates marginally stronger dispersion interactions than for Complex I, with Complex III in between these two. Overall, TAM achieves the strongest noncovalent binding, followed closely by GEN and then TQ, in line with their docking and MD stability profiles.

**Figure:**
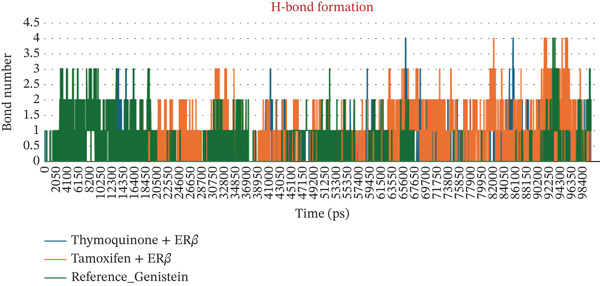
(a)

**Figure:**
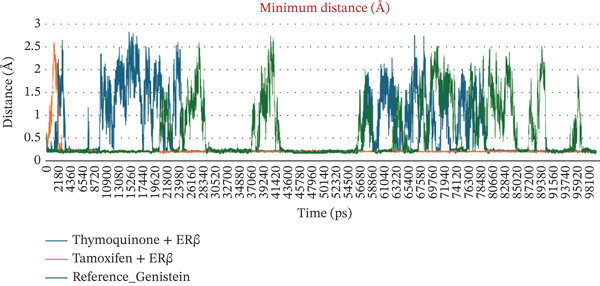
(b)

**Figure:**
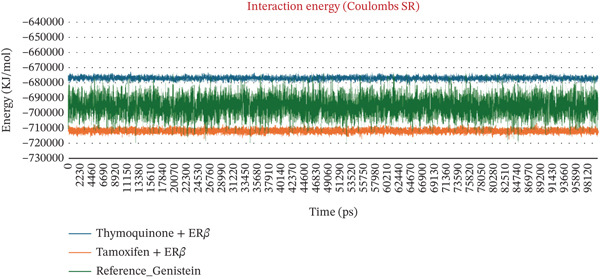
(c)

**Figure:**
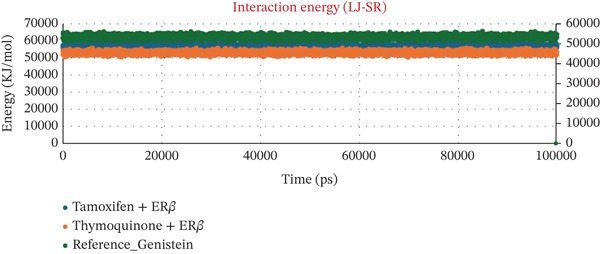
(d)

### 3.3. Cytotoxic Effect of TQ and TAM on TNBC Cells

To determine the optimized concentrations of TQ and TAM against TNBC cells, we used the MTT assay, varying concentrations of both compounds from low to high. During the screening, we observed differential effects of the compounds on cell proliferation, and the effective concentrations were determined as IC_50_ values. The cytotoxic effects of TQ and TAM, alone and in combination, were evaluated on MDA‐MB‐468 and MDA‐MB‐231 cell lines after 24 h of treatment. The results obtained after 24 h showed a dose‐dependent increase in cell cytotoxicity in both cell lines (Figure S1a,b). The IC_50_ values were estimated to be 18 ± 3.40 and 22 ± 2.90 * μ*M for MDA‐MB‐468 and MDA‐MB‐231, respectively. Treatment with TAM resulted in a considerable decrease in IC_50_, to approximately 11 ± 0.82 and 15 ± 3.89 * μ*M for MDA‐MB‐468 and MDA‐MB‐231, respectively. The average IC_50_ concentrations for TQ and TAM (~20 and 13 *μ*M, respectively) were used for further studies in both cell lines.

### 3.4. Cell Morphological Analysis

Overall, the shape and structure of both cells were assessed after treatment with TQ (20 *μ*M) and TAM (13 *μ*M) alone or in combination using F‐actin and DAPI staining. Intact morphology was observed in control cells. Treatment with TQ resulted in enhanced damage to the nucleus and cytoskeleton compared to TAM (Figure S2a) in MDA‐MB‐468 cells. However, the combination of TQ and TAM showed highly ruptured cells, as indicated by the red arrows. A similar effect was observed on MDA‐MB‐231 cells, as shown in Figure S2b. The enhanced activity of the TQ and TAM combination was noticed compared with individual treatment against both cell lines. However, the combination treatment was more effective on MDA‐MB‐468 than MDA‐MB‐231 cells, as shown in Figure S2a,b.

### 3.5. Cell Death Analysis

To study the effect of TQ, TAM alone, and in combination on cell death properties against both cell lines, calcein AM–EthD‐III staining was performed, as shown in Figure 4a,b. The green fluorescent cells indicate live cells. As calcein AM enters the cytoplasm, it is cleaved by esterases in live cells to yield the green fluorescent dye calcein. The red‐stained cells indicate dead cells because EthD‐III penetrates dead cells and stains the nucleus with bright red fluorescence. The cytotoxic effect of TQ (20 *μ*M) on MDA‐MB‐468 cells was less than that of TAM (13 *μ*M). However, their combination showed enhanced cell death, as observed in Figure 4a. In MDA‐MB‐231 cells, TQ and TAM had almost identical effects. However, their combination showed enhanced cytotoxicity, resulting in a higher number of dead cells, as shown in Figure 4b. The combination treatment was more effective and induced cell death in MDA‐MB‐468 cells than in MDA‐MB‐231 cells (Figure 4a,b).

Figure 4Effect of TQ and TAM individually or in combination on TNBC cells. Cells were treated with the average IC_50_ concentration for 24 h, and images were captured at magnification (10×) through EVOS FL. The color green represents live cells, and the red represents dead cells in MDA‐MB‐468 (a) and MDA‐MB‐231 (b).

**Figure:**
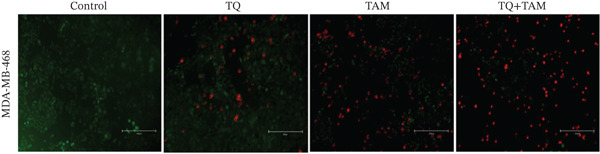
(a)

**Figure:**
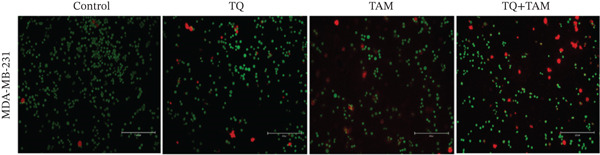
(b)

### 3.6. Assessment of Apoptosis

**Figure 5:**
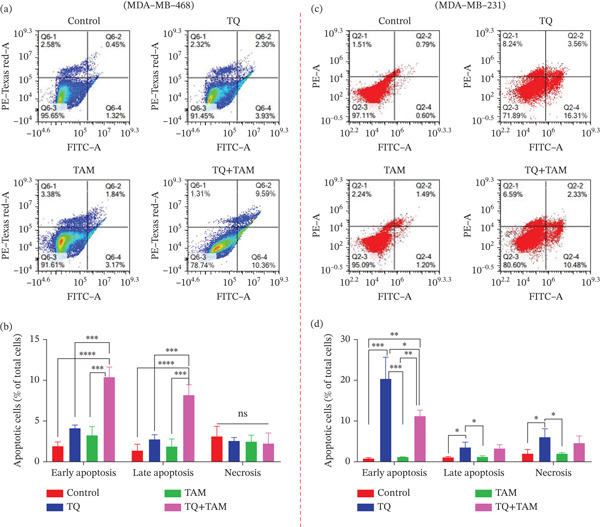
Apoptosis/necrosis assessment in TNBC cells after exposure for 24 h with TQ, TAM, and their combination. Typical apoptotic population of cells stained with annexin V‐FITC/PI and total events are presented (a) Dot plot showing apoptotic population with different treatment groups and (b) the percentage of different cell populations of total events on MDA‐MB‐468 cells. (c) Represents a dot plot of apoptotic population and (d) percentage of different cell populations of total events on MDA‐MB‐231 cells. Each bar indicates the mean ± SEM of three replicates using one‐way ANOVA. Statistical significance is considered at ∗*p* < 0.05, ∗∗*p* < 0.01, ∗∗∗*p* < 0.001, ∗∗∗∗*p* < 0.0001, and *p* > 0.05 as ns (nonsignificant).

To further confirm the cell death process (programmed or nonprogrammed) induced by TQ, TAM, and their combination, TNBC cells were stained with annexin‐FITC/PI and analyzed by flow cytometry to quantify apoptotic and necrotic cells. In MDA‐MB‐468 cells, TQ treatment showed a slightly greater apoptotic effect than TAM. However, the combination of TQ and TAM significantly enhanced apoptosis, as observed in Figure 5b. Almost 10.36% of the cells were found in the early apoptotic phase and 9.59% cells in the late apoptotic phase, as observed in Figure 5a,b. The effect of individual treatment or in combination showed nonsignificant change in necrotic properties. In MDA‐MB‐231 cells treated with TQ alone, a higher number of apoptotic cells was observed in both the early and late phases compared with those treated with TAM, as shown in Figure 5c,d. Though, the combination of TQ and TAM showed significantly enhanced apoptosis compared to TAM alone but not to a level higher than that of TQ alone (Figure 5d).

### 3.7. Wound Healing

Next, we elucidated the effect of combination therapy on cell migration in MDA‐MB‐468 and MDA‐MB‐231 cells. The scratch wound assay was performed to compare the effects of TQ and TAM alone and their combination on the delay in wound healing, as presented in Figure 6a,c for both cell lines. The percentage wound area was estimated for both cells through ImageJ software and presented in Figure 6b,d. The wounds of both control cells (untreated) were reduced significantly after 24 h of incubation, as depicted in Figure 6a,c. However, TQ showed significant antiwound‐healing properties compared with the control in MDA‐MB‐468 cells (Figure 6b). Similar results were observed in MDA‐MB‐231 cells treated with TQ and TAM, as presented in Figure 6c,d. The combination effect on antiwound‐healing activity against MDA‐MB‐468 cells was higher than that of MDA‐MB‐231 cells at 24 and 48 h (Figure 6b). In short, the current combination treatment was more effective against MDA‐MB‐468 cells.

Figure 6Combination treatment inhibits the cell migration and invasion of TNBC cells. Wound‐healing assay showed the effects of TQ, TAM, and TQ + TAM to prevent wound closure at IC_50_ concentrations of individual and their combination. (a) Image showing wound area at different time points after drug treatment, (b) represents bar graph of percentage wound area in MDA‐MB‐468 cells, (c) depicts image of wound area captured at different time point for different treatment group, and (d) represents bar graph of percentage wound area in MDA‐MB‐231 cells. Representative images were captured at the indicated time points after drug treatment for both cell lines. The percentage of the wound area of each treatment group was compared with its counterparts at the start point (0 h). *p* values were determined relative to the control group. Significant values were considered at ∗*p* < 0.05, ∗∗*p* < 0.01, ∗∗∗*p* < 0.00, and *p* > 0.05 as ns (nonsignificant).

**Figure:**
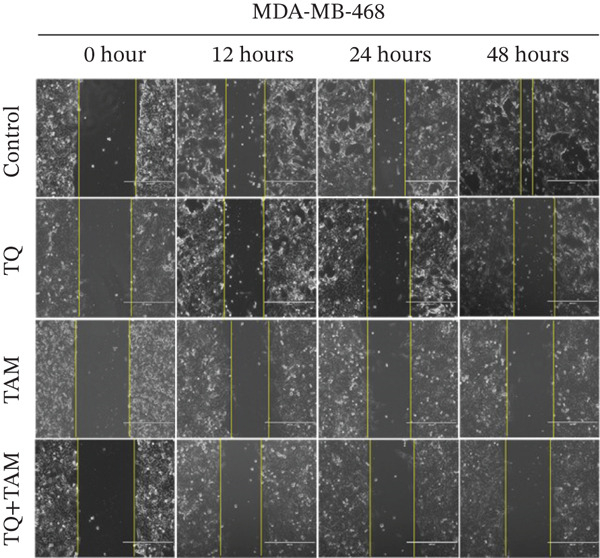
(a)

**Figure:**
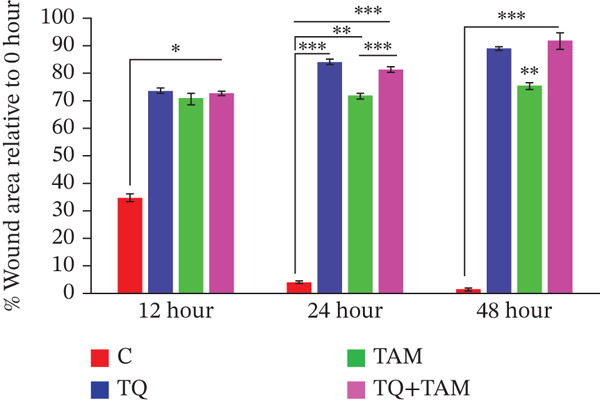
(b)

**Figure:**
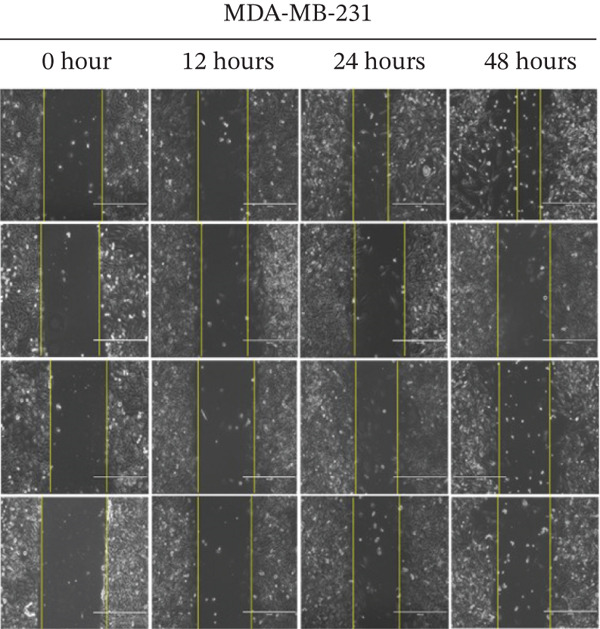
(c)

**Figure:**
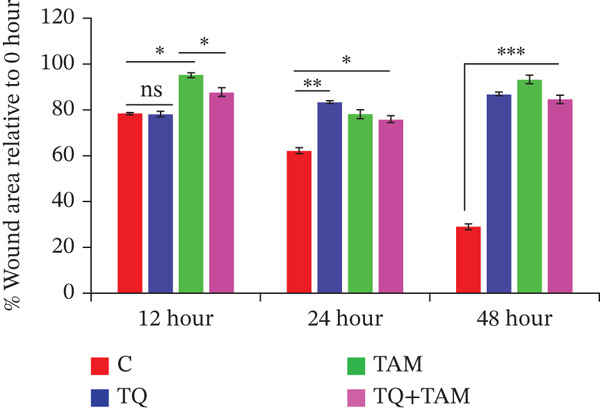
(d)

### 3.8. Cell Adhesion Analysis

Understanding cell adhesion is crucial as it plays a critical role in tumor growth, invasion, and metastasis. By elucidating cell adhesion, we can gain deeper insights into the interaction between cancer and its environment, which will help develop targeted therapies to intervene in this interaction and inhibit cancer progression. The change in adhesion properties was continuously recorded using an accurate real‐time cell analysis (RTCA) instrument for 24 h. After treatment with TQ, TAM, or their combination, cell adhesion was altered in Figure 7a,c for both cell lines. A significant inhibition of cell adhesion was observed at a specific time point, 20 h, across all treatments as shown in the bar graphs in Figures b and d for both cell lines. TQ showed significant inhibition of cell adhesion compared to TAM in both cell lines, as shown in Figure 7b,d. However, the combination treatment showed significantly enhanced inhibition compared to individual therapy against both cell lines.

Figure 7The inhibitory effect of TQ and TAM alone or in combination impacts cell adhesion properties. The adhesion properties over the period are presented for (a) MDA‐MB‐468 and (c) MDA‐MB‐231 cells. The bar graphs illustrate the inhibitory effects of treatments at approximately 20 h on MDA‐MB‐468 and MDA‐MB‐231 cells, respectively (b, d). Each bar indicates the mean ± SEM of three replicates. Statistical significance is considered at ∗*p* < 0.05, ∗∗*p* < 0.01, ∗∗∗*p* < 0.001, and *p* > 0.05 as ns (nonsignificant).

**Figure:**
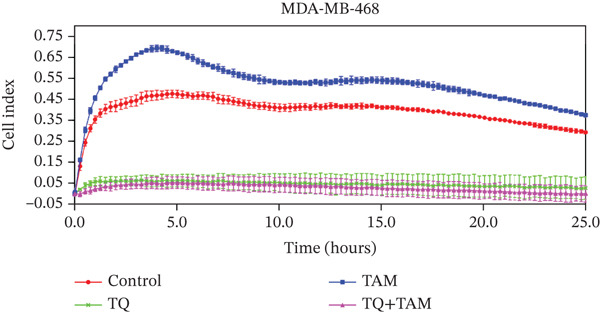
(a)

**Figure:**
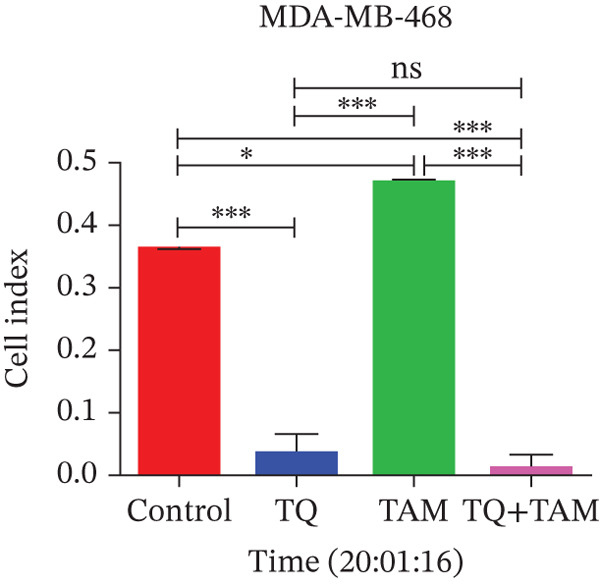
(b)

**Figure:**
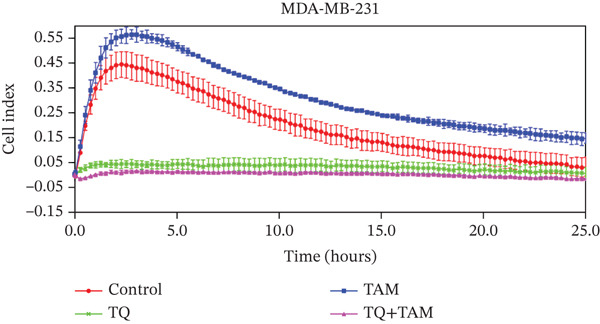
(c)

**Figure:**
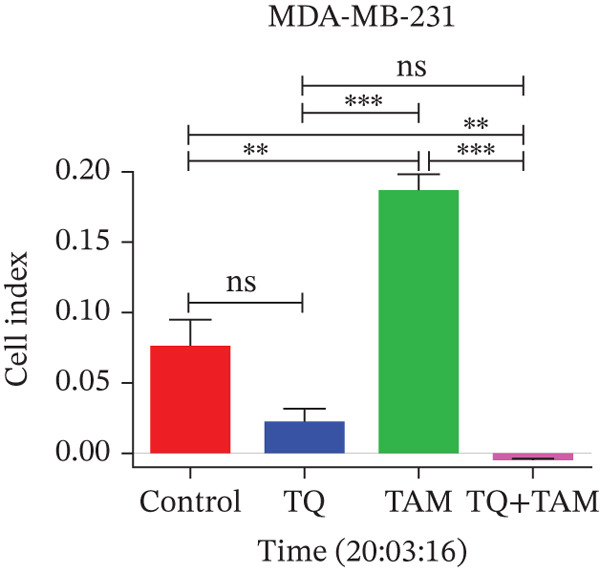
(d)

### 3.9. EMT‐Associated Gene Expression Alteration in Treated TNBC Cells

**Figure 8:**
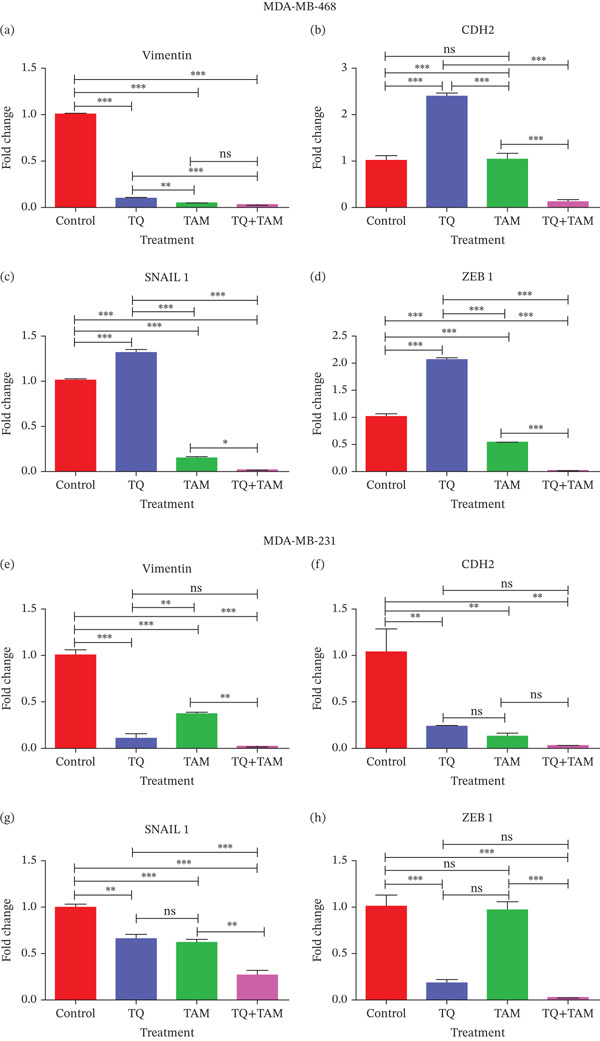
The effects of TQ and TAM on the expression of genes involved in EMT in TNBC were evaluated in MDA‐MB‐468 and MDA‐MB‐231 cells. The difference in mRNA expression between the treatments and the control was used to calculate gene expression. Bar graphs show the expression of different target genes in MDA‐MB‐468 cells (a–d). Bar graphs represent the expression of other target genes in MDA‐MB‐231 cells (e–h). The findings illustrate the means of three replicates. Using ANOVA in one‐way analysis, the statistical significance of the differences between control and treatments was established. The statistically significant differences were indicated by ∗*p* < 0.05, ∗∗*p* < 0.01, ∗∗∗*p* < 0.001, and ns: *p* > 0.05.

Treated cells were subjected to RNA isolation, cDNA synthesis, and quantitative PCR (qPCR) to evaluate EMT‐associated gene expression and examine the compounds′ anticancer effects. Both TNBC cell lines were treated with TQ (20 *μ*M), TAM (13 *μ*M) individually, and in combination. The expression profile of different target genes in MDA‐MB‐468 cells is presented in Figures 8a, 8b, 8c, and 8d. Vimentin expression levels are remarkably reduced in all treatment groups. The expression of an essential mesenchymal marker, N‐cadherin, was significantly lower in the combinational treatment compared with the control.

However, a significant decrease in combination indicates an enhanced inhibitory effect on EMT (Figure 8b). A similar trend was observed for another EMT core regulator, SNAIL1, which was significantly downregulated in the combination group (Figure 8c). Reduced vimentin expression also plays a vital role in regulating transcription factor ZEB1, which is decreased in the combination group (Figure 8d). The expression of vimentin was significantly reduced in MDA‐MB‐231 cells treated with TQ compared with those treated with TAM alone. However, TQ + TAM showed significant downregulation of vimentin (Figure 8e). The expression of N‐cadherin was also significantly downregulated upon treatment individually and in combination, as shown in Figure 8f. A significant decrease in SNAIL1 and ZEB1 expression was also observed in the combination treatment presented in Figure 8g,h.

### 3.10. TQ and TAM Combination Reduces the EMT and Cell Adhesion Proteins

Furthermore, we investigated the expression of vimentin and vinculin proteins, which are associated with EMT, migration, and increased invasiveness in TNBC. The suppressive effects of TQ and TAM, as well as their combination, on protein expression were evaluated using western blot analysis after 24 h of administration against both TNBC cell lines represented in Figure 9a,e as cropped bands of the full blot supplied in Figure S3 for all studied markers in this study. Densitometry was performed to quantify protein expression, as shown in the bar graphs. The data were normalized with GAPDH as an internal control. Treatment with TQ and TAM alone showed no significant decrease in vimentin level in MDA‐MB‐468 cells (Figure 9b). However, treatment in combination (TQ + TAM) significantly reduced the level of vimentin. Treatment with TQ alone did not inhibit vinculin protein expression. However, TAM alone and combined with TQ exhibited significantly reduced vinculin levels (Figure 9c). The efficacy of treatment on E‐cadherin expression was also examined. The treatment with TQ and TAM alone did not upregulate E‐cadherin. However, a significant increase in E‐cadherin levels was observed with combination treatment of TQ + TAM (Figure 9d). A significant reduction in vinculin level was also observed across different treatment groups. In the case of MDA‐MB‐231 cells, TQ and TAM alone as well as in combination showed significantly reduced levels of vimentin (Figure 9f). The combination treatment showed greater inhibition than individual therapy. A significant decrease in vinculin levels is also observed with TQ and TAM alone compared with the control. However, the combination of TQ and TAM shows a significant decrease in vinculin levels compared with the control and other individual treatment groups (Figure 9g). Treatment with TQ and TAM alone did not show a significant increase in E‐cadherin levels. However, a significant increase in E‐cadherin level is observed in the combination treatment of TQ + TAM (Figure 9h).

Figure 9Western blot analysis results. Blots showed vimentin, vinculin, and E‐cadherin expression in (a) MDA‐MB‐468 and (e) MDA‐MB‐231 cells treated with TQ, TAM, and TQ + TAM. Control cells were exposed to 0.1% DMSO. The bar graphs depict the western blot quantification for the same treatments in MDA‐MB‐468 (b–d) and MDA‐MB‐231 (f–h). The fold change was estimated by measuring density normalized with GAPDH using ImageJ software. Data generated from three independent experiments were evaluated for statistical significance effects ∗*p* < 0.05, ∗∗*p* < 0.0, ∗∗∗*p* < 0.001, and ns: *p* > 0.05.

**Figure:**
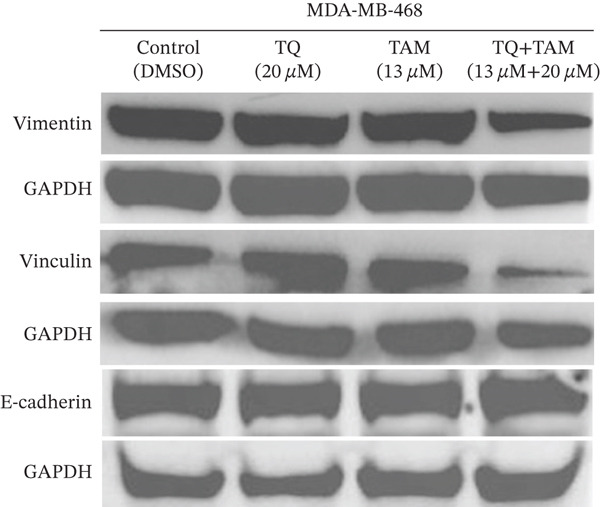
(a)

**Figure:**
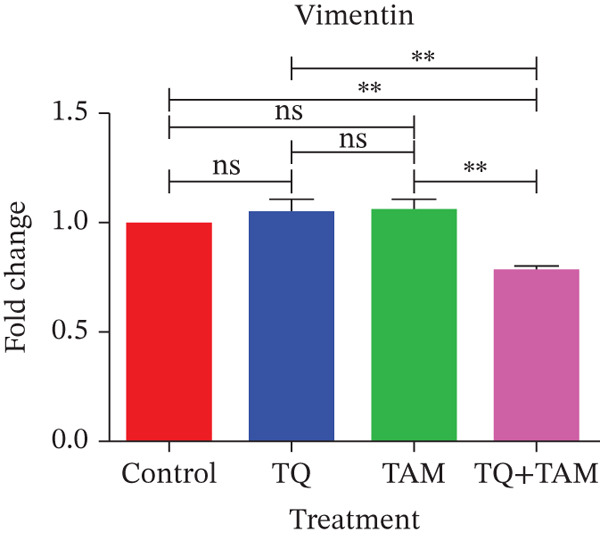
(b)

**Figure:**
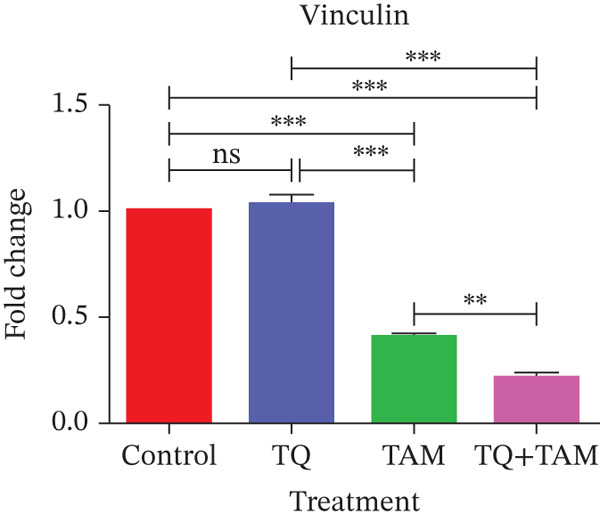
(c)

**Figure:**
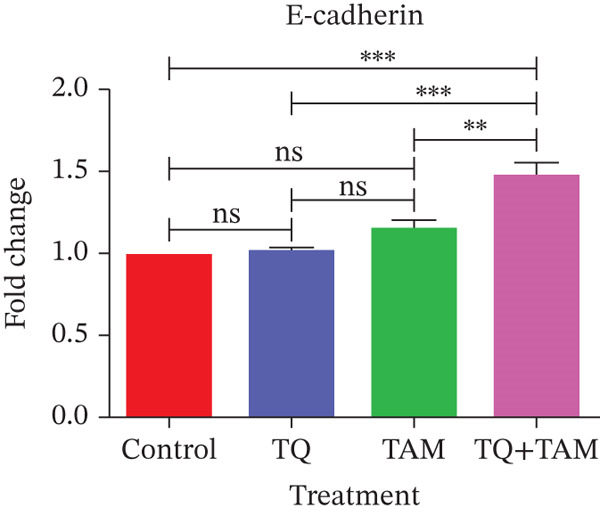
(d)

**Figure:**
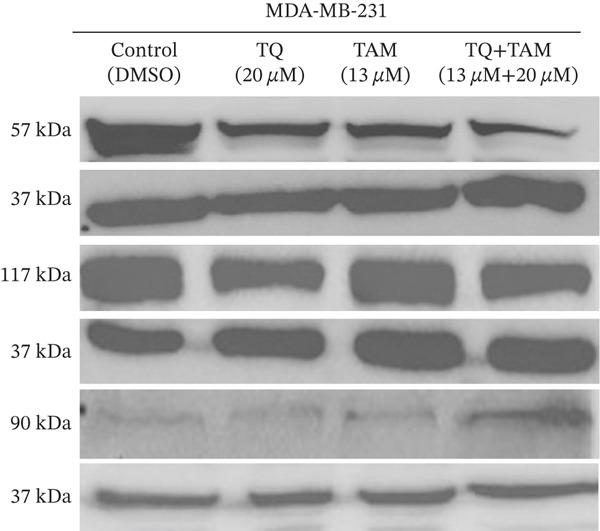
(e)

**Figure:**
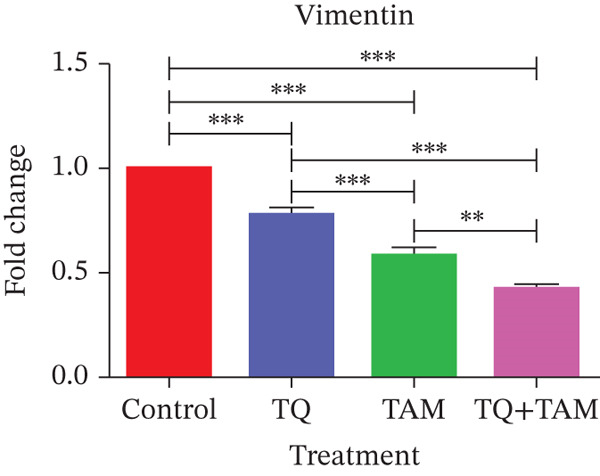
(f)

**Figure:**
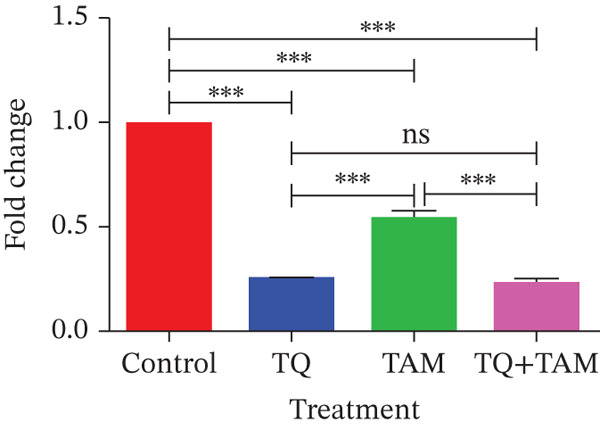
(g)

**Figure:**
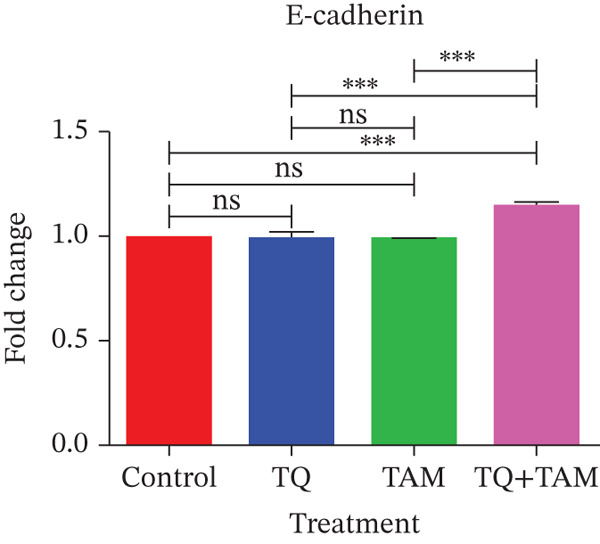
(h)

## 4. Discussion

Targeted therapies for TNBC are limited; conventional chemotherapy and radiotherapy are mainly used to treat breast cancer patients [48]. These therapies are not specific and fail to target resistant cells [49]. TAM has been widely accepted as a therapeutic for early and advanced estrogen‐positive breast cancer [50]. TAM has demonstrated significant anticancer potential, primarily in ER‐positive breast cancers. Different strategies are employed to mitigate side effects in patients, with lower doses also found to be as effective as higher doses [51]. However, its effectiveness against TNBC is limited due to the lack of ERs, the primary target of TAM. However, a recent study suggests that TAM may have some antitumor effects in TNBC, possibly by modulating the estrogen‐related tumor microenvironment or interacting with the ER*β* receptor [52]. The TAM activity can be regained through a combinatorial approach, as evidenced by specific reports [13, 14]. The ER*β* expression in TNBC provides a rationale for choosing TAM for TNBC treatment [52]. Additionally, TQ alone exhibits significant antitumor effects, including in TNBCs [53].

The in silico analysis demonstrated favorable binding affinities and stable ligand–receptor interactions, suggesting that ER*β* could be a potential target receptor for TQ and TAM combination therapy. TQ showed higher binding affinities than TAM through the key residues such as Leu343, Phe356, and Ala302, indicating potential inhibitory conformational changes within the receptor. The in silico studies served as the basis for further in vitro anticancer assay validations.

Abdelmaleket et al. recently reported gefitinib‐TAM hybrid ligands and found excellent anticancer properties against breast cancer cells, especially TNBC (MDA‐MB‐231 and MDA‐MB‐468) cells [54]. In our initial studies, we observed an improved TAM efficacy in the presence of TQ, significantly elevated cytotoxicity level from 15% to 75% in the combination against both cell lines. In addition, these results were supported by alterations in morphological and cytoskeletal parameters, leading to cytotoxicity. Further significant increased apoptosis confirms the combinatorial efficacy and provides evidence of the regaining potential of TAM. Considering TNBC cells′ metastatic potential, it was important to determine whether these combinations affect EMT. In addition, cell adhesion to neighboring cells and the underlying ECM is required for cell survival and plays a vital role in tumor migration [55]. The treatment with combinations of TQ and TAM led to extensive alterations in cell adhesion and reduced vinculin levels, suggesting disruption of focal adhesions and a reduction in migratory potential. The wound recovery percentages in the combination treatment group were significantly reduced in both TNBC cell lines. Further, at the molecular level, reduced vimentin and N‐cadherin (CDH2) and a concomitant increase in E‐cadherin were observed in the combination treatment in both cell lines, confirming the antimetastatic effect. Based on some previously reported findings, ER*β* signaling can interfere with TGF‐*β*/SMAD and PI3K/ERK pathways, reducing EMT [56] [57, 58]. In addition, ER*β* activation can reduce the transcription factors SNAIL1 and ZEB1, which promote EMT by decreasing E‐cadherin and increasing vimentin and N‐cadherin [59–61]. These EMT markers are essentially involved in the TNBC EMT process, and our study outcomes lead to anticancer and antimetastatic potential possibly driven through ER*β* signaling that needs further validation.

The transcription factors SNAIL1 and ZEB1 (core regulators of EMT) bind to the E‐cadherin (CDH1) promoter, downregulate its expression, and overexpress N‐cadherin, leading to cell migration [62, 63]. Vimentin regulates the expression of SNAIL1 and ZEB1, and vimentin inhibition reduces their expression [64]. The combination of TQ and TAM showed a promising inhibitory effect on the expression of EMT‐associated genes (vimentin, N‐cadherin, SNAIL1, and ZEB1), resulting in an antimetastatic effect in TNBC cells. Similarly, inhibition of N‐cadherin prevents the expression of SNAIL1 and ZEB1, thereby inhibiting EMT [65]. The combinatorial approach reduced migration and reversed the mesenchymal traits of TNBC cells, restoring epithelial traits. TAM itself exhibits cytotoxicity to normal cells, which could be a concern; however, using TQ, which exhibits very potent cytotoxicity, allowed for a lower dose of TAM in the combination to significantly reduce TNBC growth and metastasis, thereby lowering the potential cytotoxicity to normal healthy cells. Given the in vitro efficacy, future studies can directly examine ER*β* involvement using receptor modulation or knockdown approaches to validate these findings in in vivo TNBC models.

## 5. Conclusion

Combination therapeutic strategies have garnered considerable attention in targeting various types of cancer. In our study, a combination of TQ and TAM demonstrated promising therapeutic potential against TNBC cell lines, despite TAM′s limited efficacy in TNBC due to the absence of hormonal receptors, which reduces its activity. The in silico docking, MD, and MD simulation results provide initial evidence for the TQ and TAM combination, with a potential interaction with ER*β*, a possible TNBC target that requires further validation. From the in vitro study, TQ appeared to enhance cytotoxic and apoptotic effects of TAM and significantly reduce cell migration, adhesion, and EMT in TNBC. The downregulation of key EMT‐associated markers, including vimentin, N‐cadherin, SNAIL1, and ZEB1, underscores the potential of this combination to suppress tumor progression and metastasis.

## Author Contributions

M.H.: conceptualization, investigation, formal analysis, original draft & editing; R.K.S.: investigation, formal analysis and editing; S.S.: investigation, analysis, writing and editing; H.W.: editing; M.K.M.: conceptualization, funding acquisition, project administration, writing original draft, review & editing.

## Funding

This study was supported by National Institutes of Health (10.13039/100000002, R25AG070244) and American Cancer Society (10.13039/100000048, DICRIDG‐22‐1037199‐01).

## Ethics Statement

The authors have nothing to report.

## Conflicts of Interest

The authors declare no conflicts of interest.

## Supporting information


**Supporting Information** Additional supporting information can be found online in the Supporting Information section. Figure S1 Cytotoxic effect of TQ and TAM on (a) MDA‐MB‐468 (b) MDA‐MB‐231 cells and IC50 determination. Figure S2 Effect of TQ and TAM individually and in combination on morphology of (a) MDA‐MB‐468 (a) MDA‐MB‐231 cells. Figure S3 Uncropped blot of Vimentin and Vinculin and E‐cadherin. Table S1 List of primers used to amplify the target gene using q‐PCR. The EMT pathway primer sequences (forward and reverse) and amplicon size are provided in the table.

## Data Availability

The data that support the findings of this study are available from the corresponding author upon reasonable request.
