# Supplementary material for: Thymoquinone Enhances Tamoxifen Efficacy Against Triple‐Negative Breast Cancer by Targeting EMT Signaling
Source: Int J Breast Cancer. 2026 Apr 6;2026:1780945. doi: 10.1155/ijbc/1780945 (PMC13053650; doi:10.1155/ijbc/1780945)
Supplement: Supplementary file 1 — Supporting Information Additional supporting information can be found online in the Supporting Information section. Figure S1 Cytotoxic effect of TQ and TAM on (a) MDA‐MB‐468 (b) MDA‐MB‐231 cells and IC50 determination. Figure S2 Effect of TQ and TAM individually and in combination on morphology of (a) MDA‐MB‐468 (a) MDA‐MB‐231 cells. Figure S3 Uncropped blot of Vimentin and Vinculin and E‐cadherin. Table S1 List of primers used to amplify the target gene using q‐PCR. The EMT pathway primer sequences (forward and reverse) and amplicon size are provided in the table. [file IJBC-2026-1780945-s001.docx]

**Thymoquinone enhances tamoxifen efficacy against triple-negative breast cancer by targeting EMT signaling**

Mazharul Haque, Ritis K. Shyanti, Sudhanshu Sharma and Manoj K. Mishra^*^

Cancer Research Center, Biological Sciences, Alabama State University, Montgomery, Alabama 36104, USA (***Corresponding Author**: Email: mmishra@alasu.edu


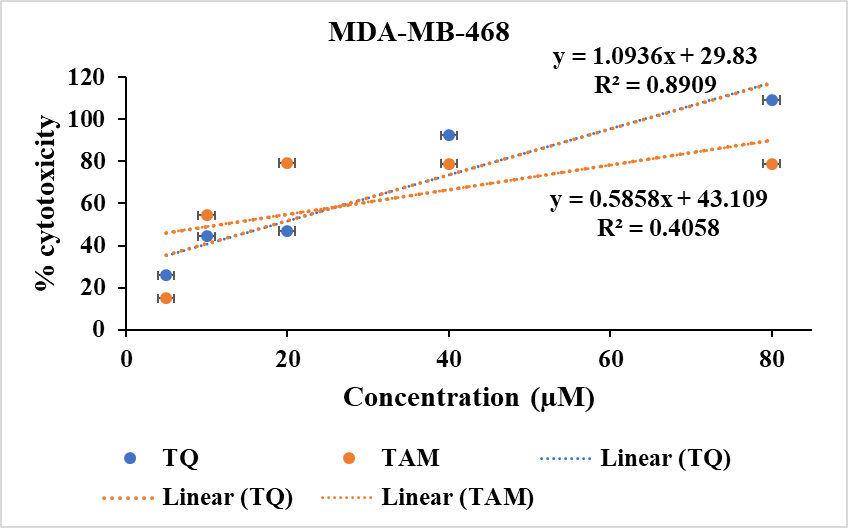


**(A)**

**
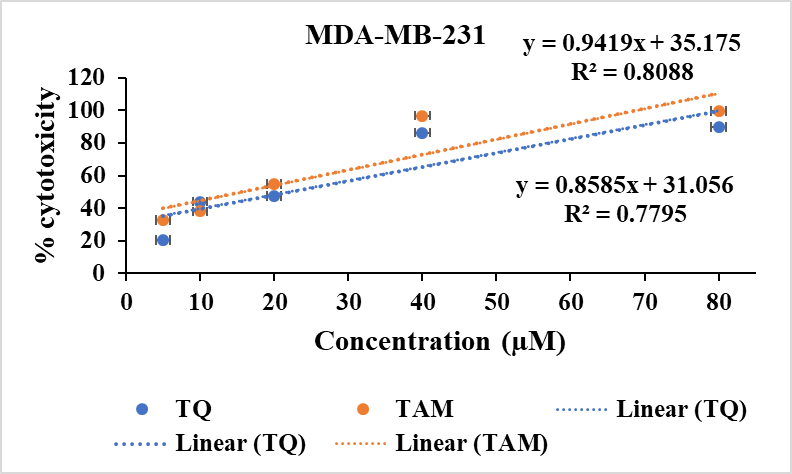
**

**(B)**

**Supplementary Fig S1:** Cytotoxic effect of TQ and TAM on (A) MDA-MB-468 (B) MDA-MB-231 cells. Determination of IC50 by estimating slope of the curve between drug concentration and % cell cytotoxicity.

**
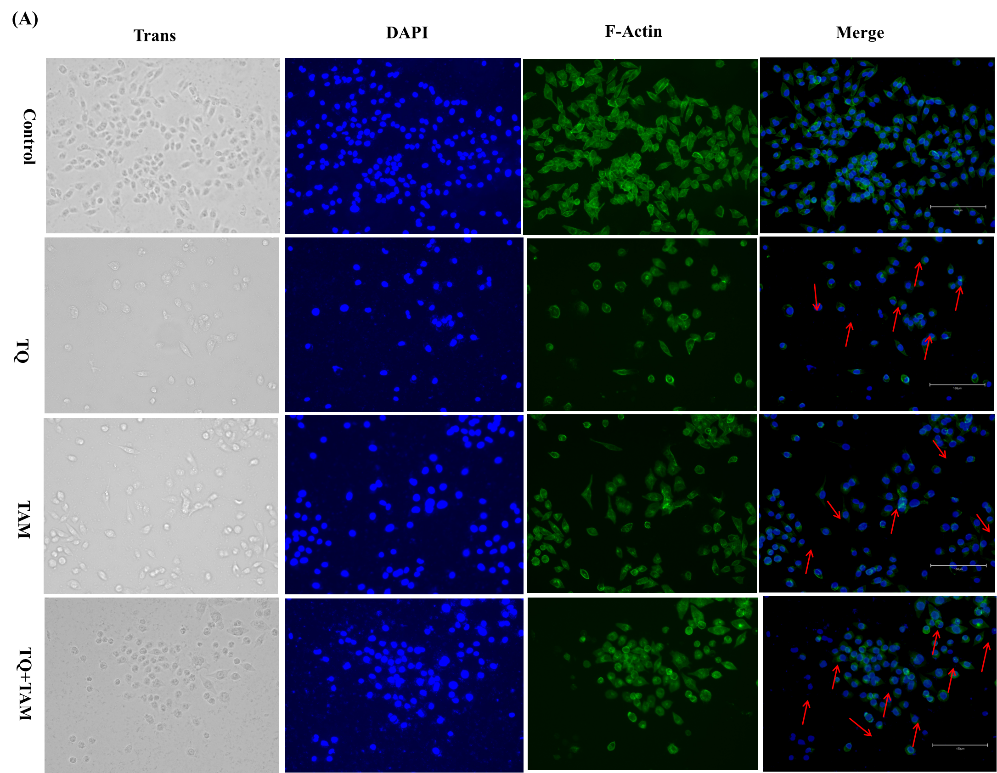
**

**
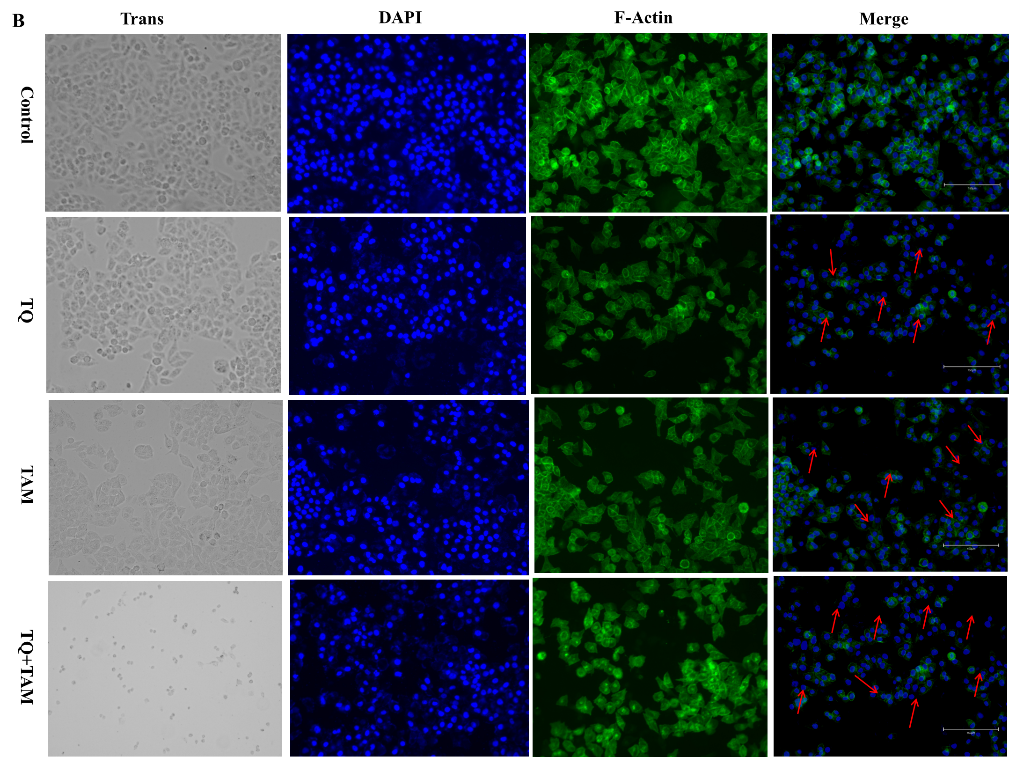
**

**Supplementary Fig. S2:** Effect of TQ and TAM individually and in combination on morphology of (A) MDA-MB-468 (B) MDA-MB-231 cells. Cells were treated with IC_50_ concentration and incubated for 24 hours, and images were captured at magnification (20X) through EVOSTM FL. (blue color represents the stained nucleus and the green color stains Actin of the cytoskeleton.

**Supplementary Fig. S3:** Uncropped blot of Vimentin and Vinculin and E-cadherin after treatment with TQ, TAM and their combination on MDA-MB-468 cells.


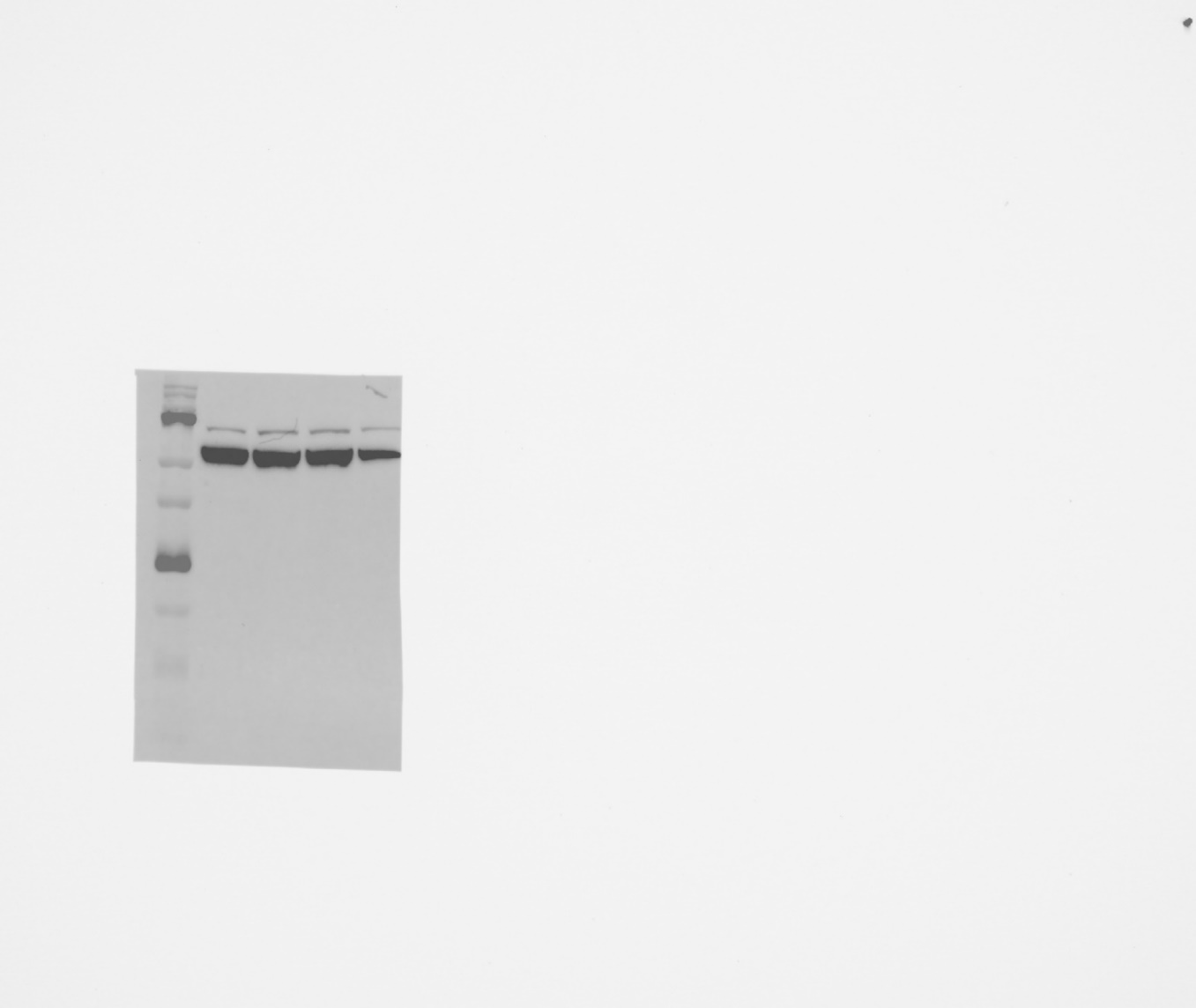

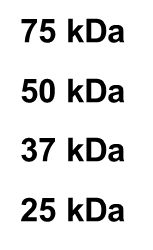

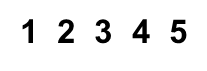

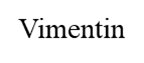

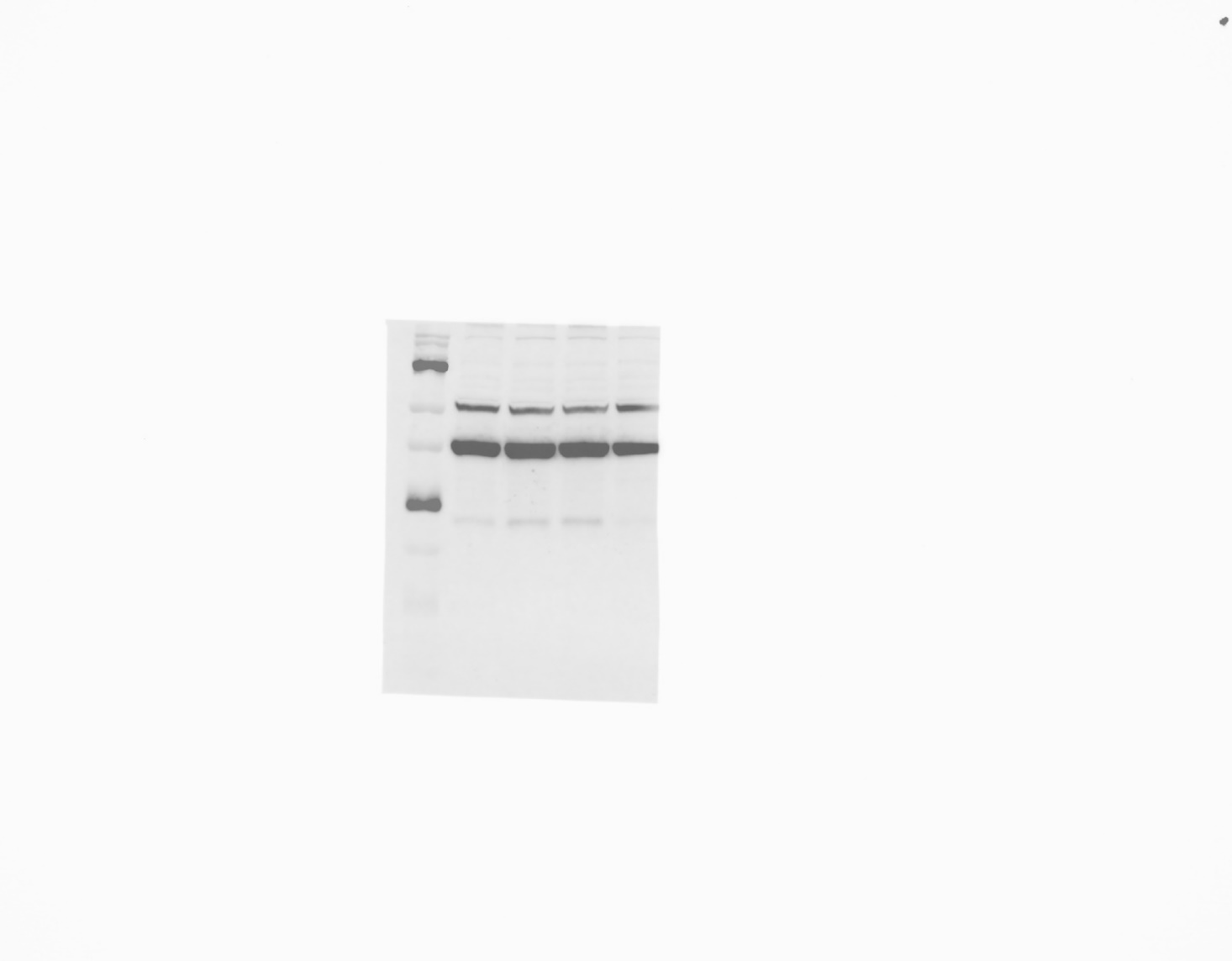

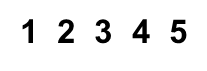

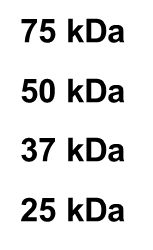

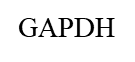

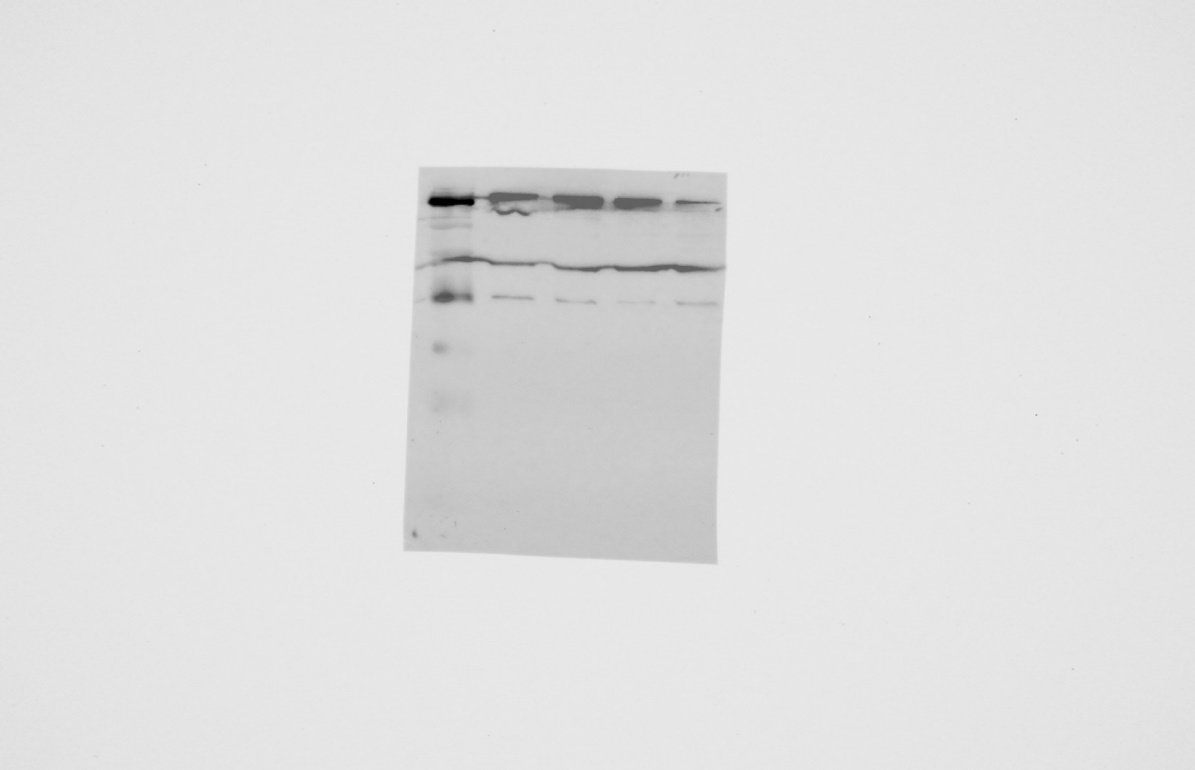

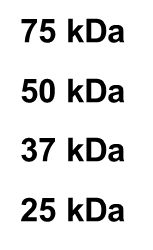

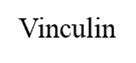

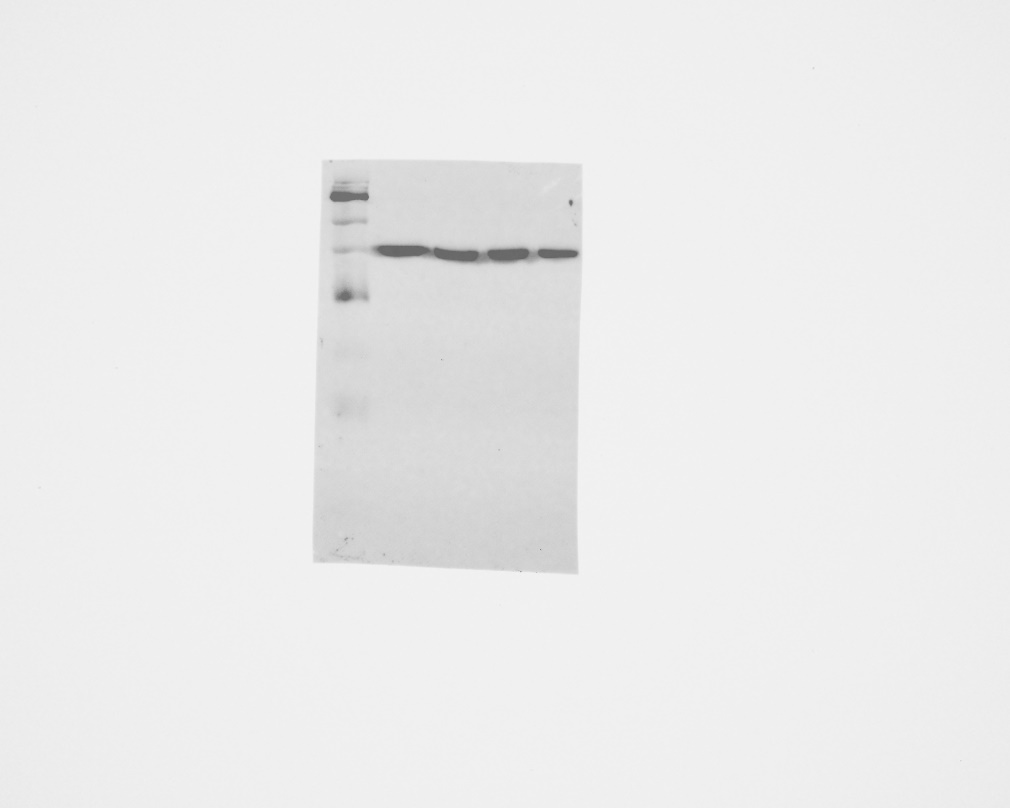

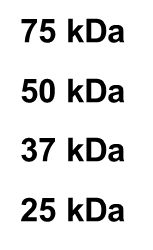

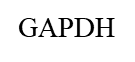


**
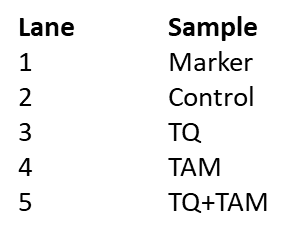
**


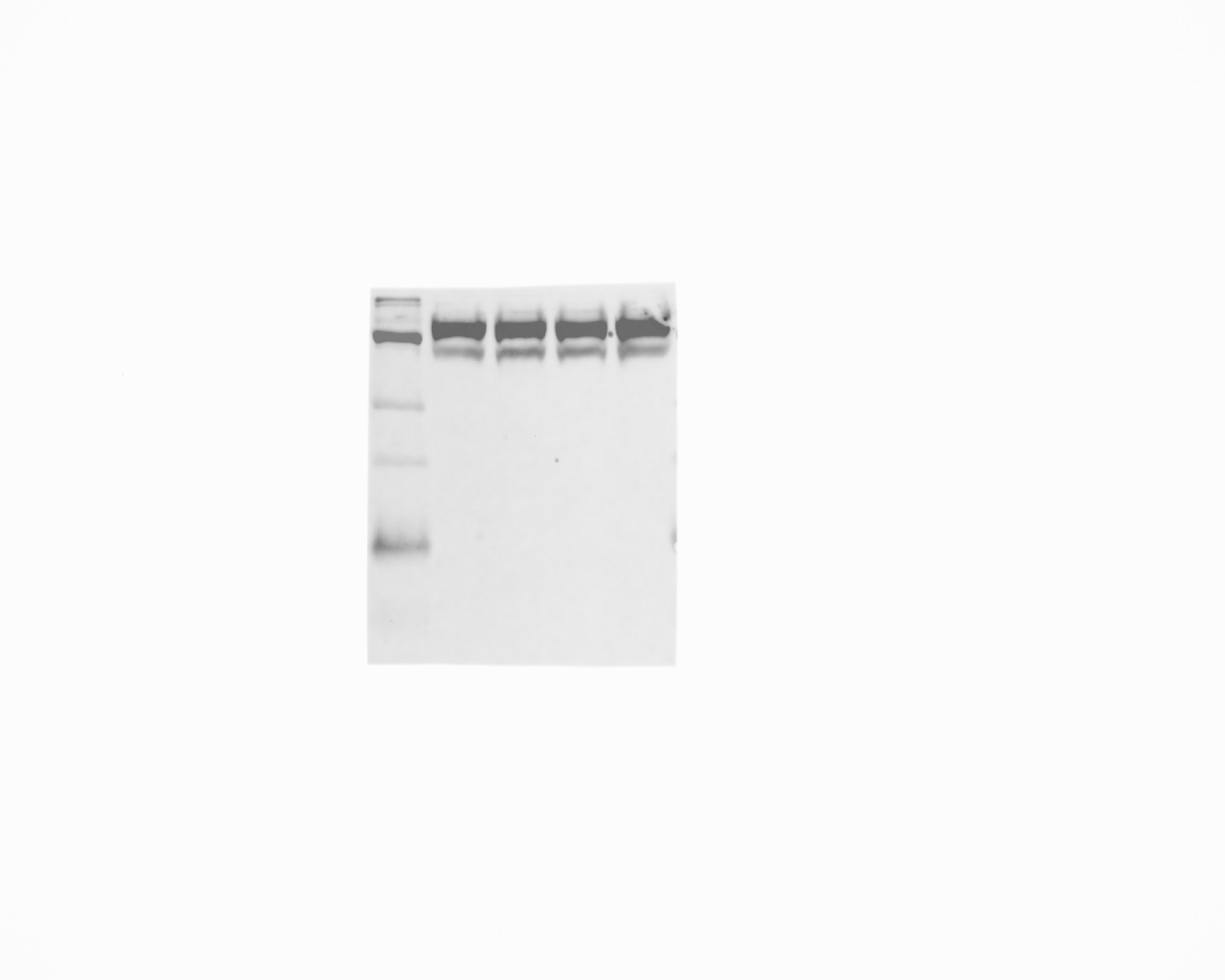

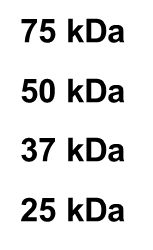


E-cadherin


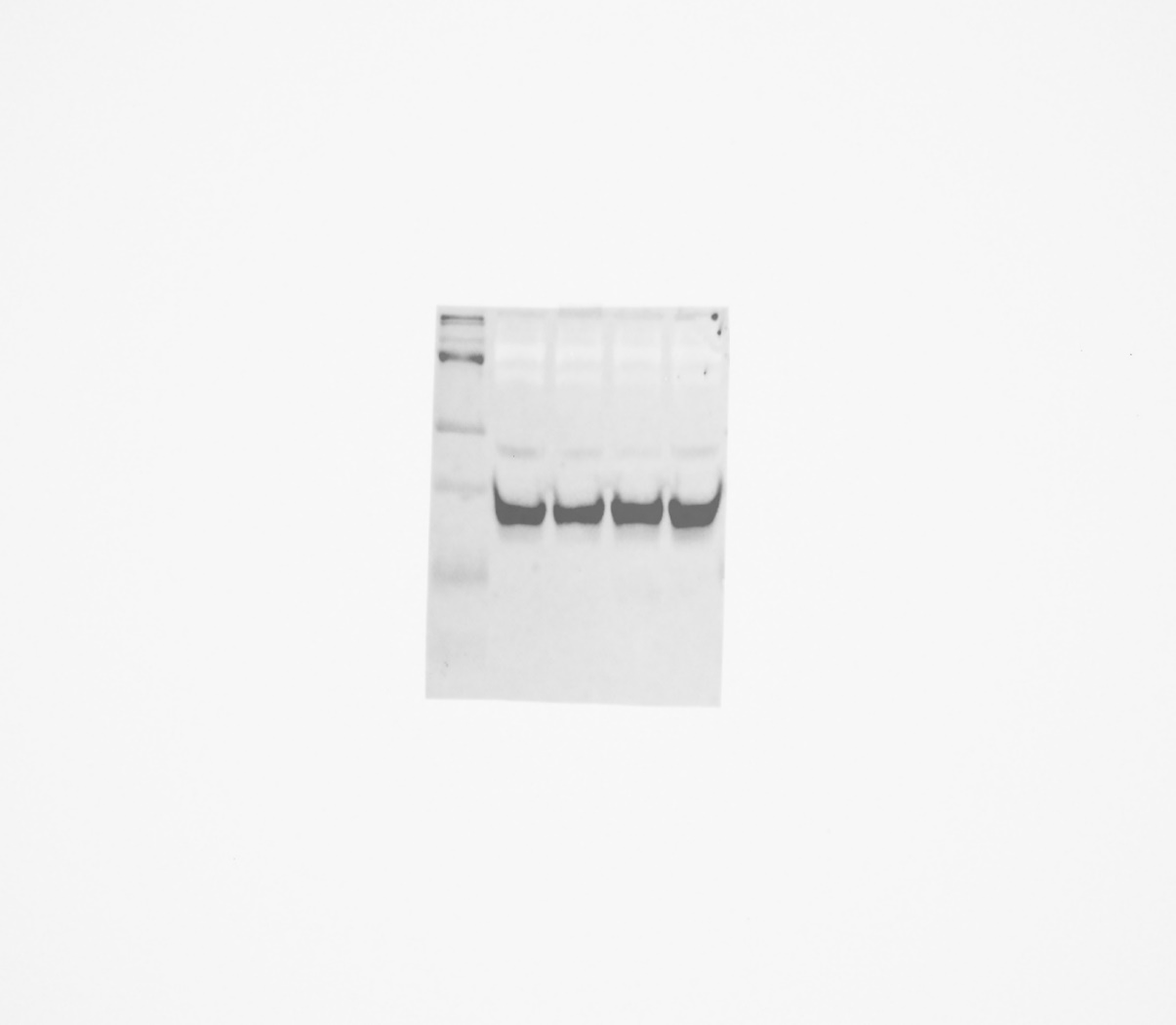


GAPDH


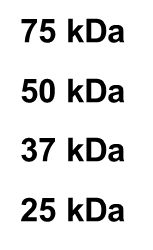


Uncropped blot of Vimentin and Vinculin and E-cadherin after treatment with TQ, TAM and their combination on MDA-MB-231 cells.


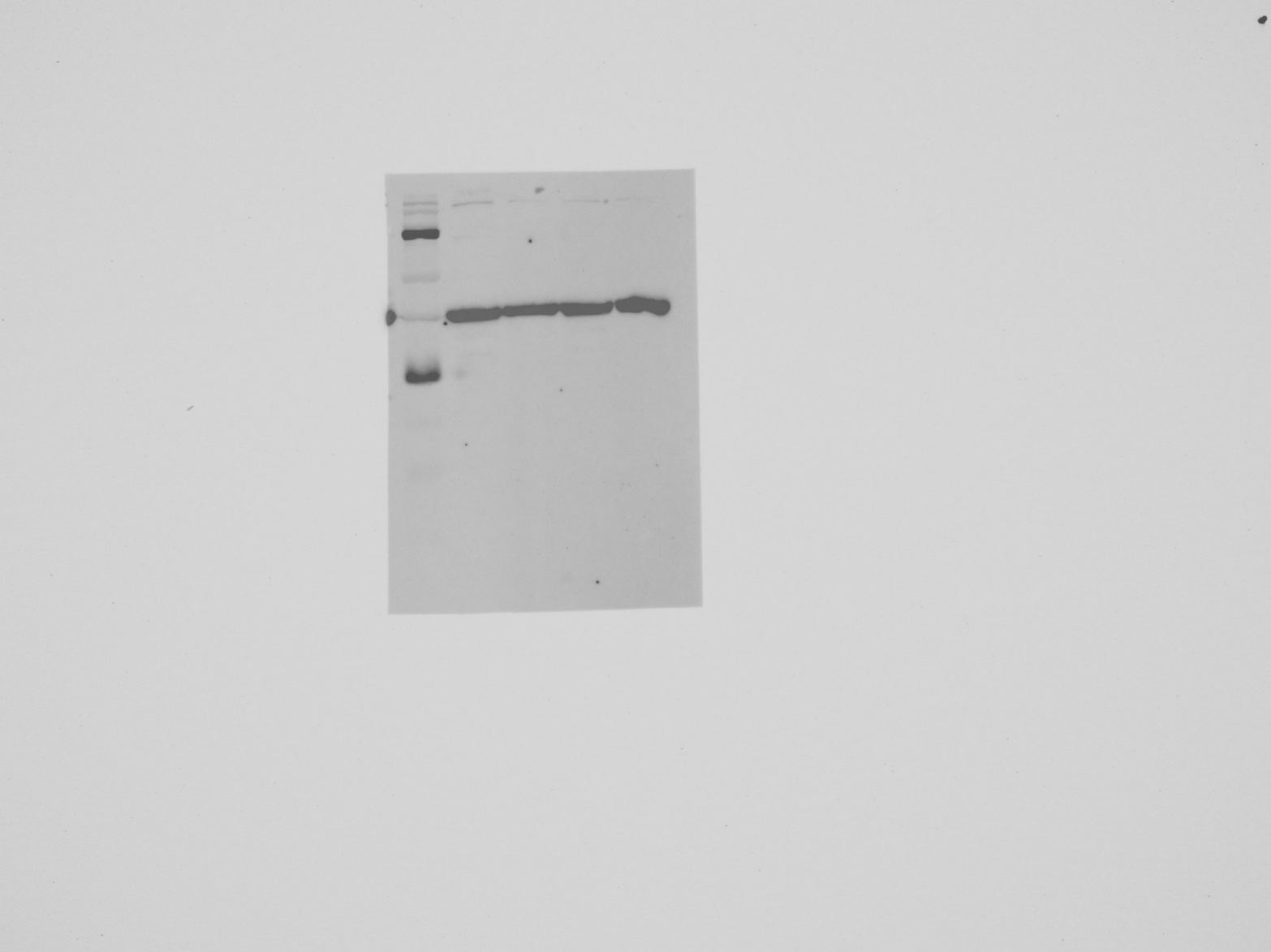

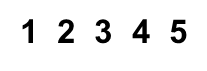

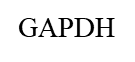

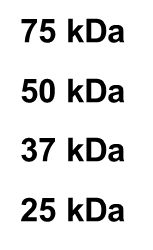

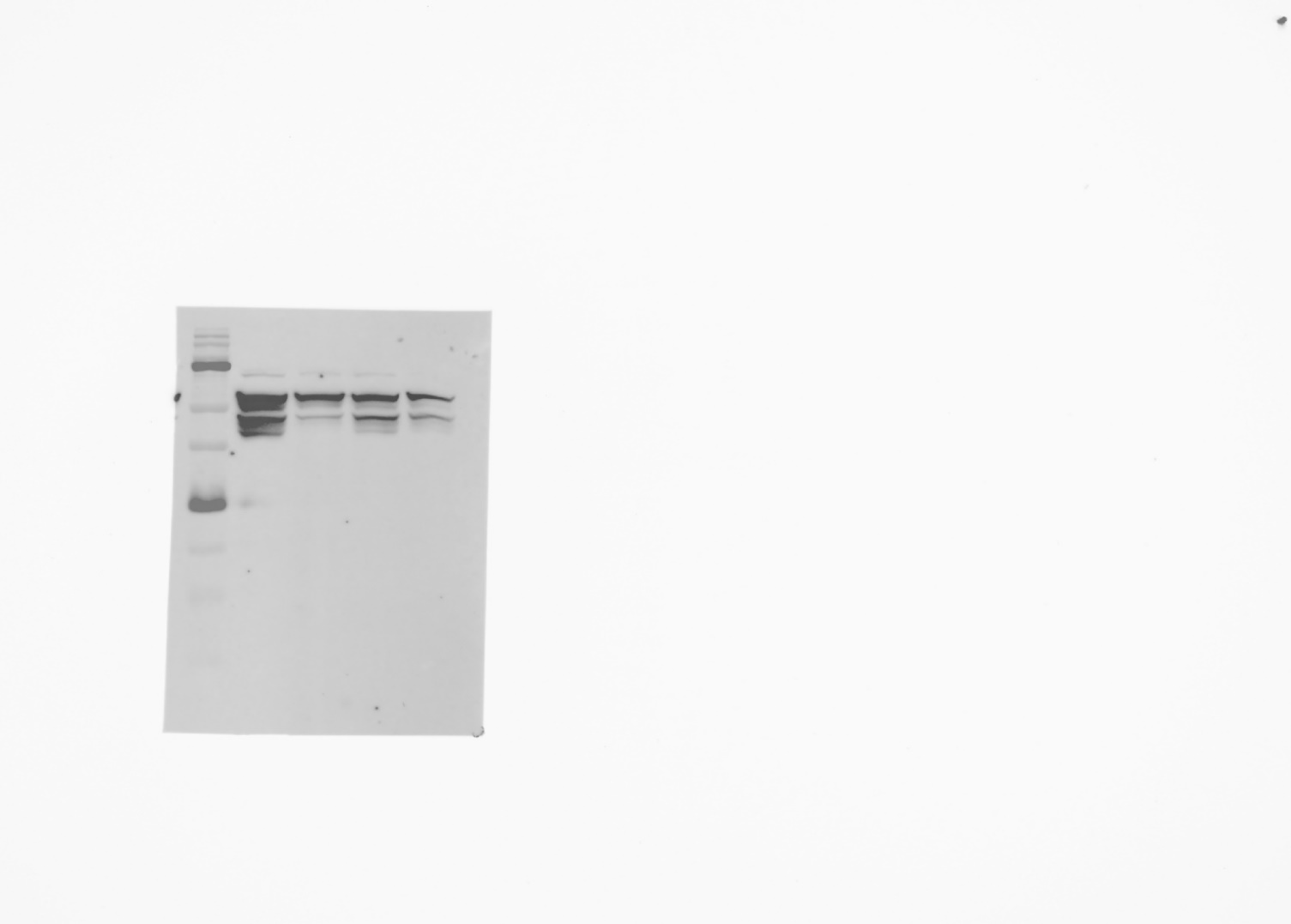

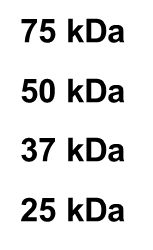

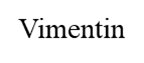

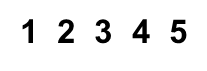

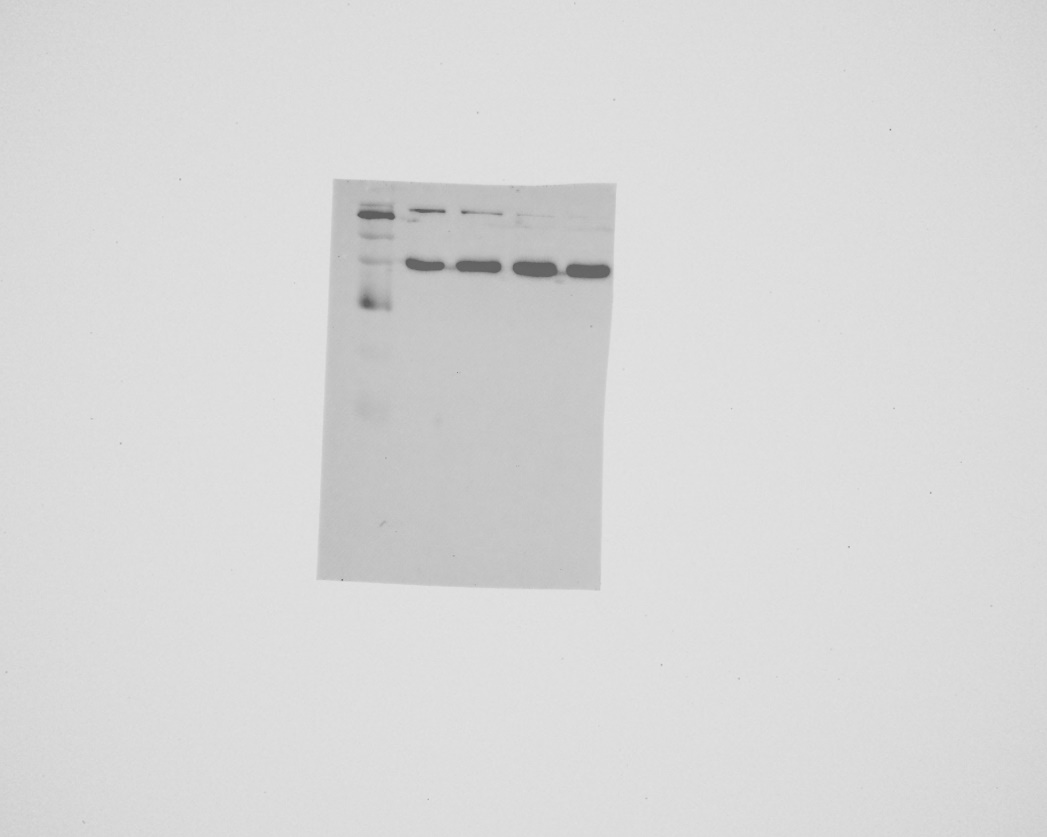

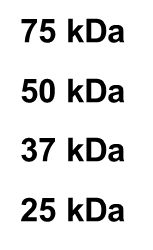

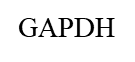

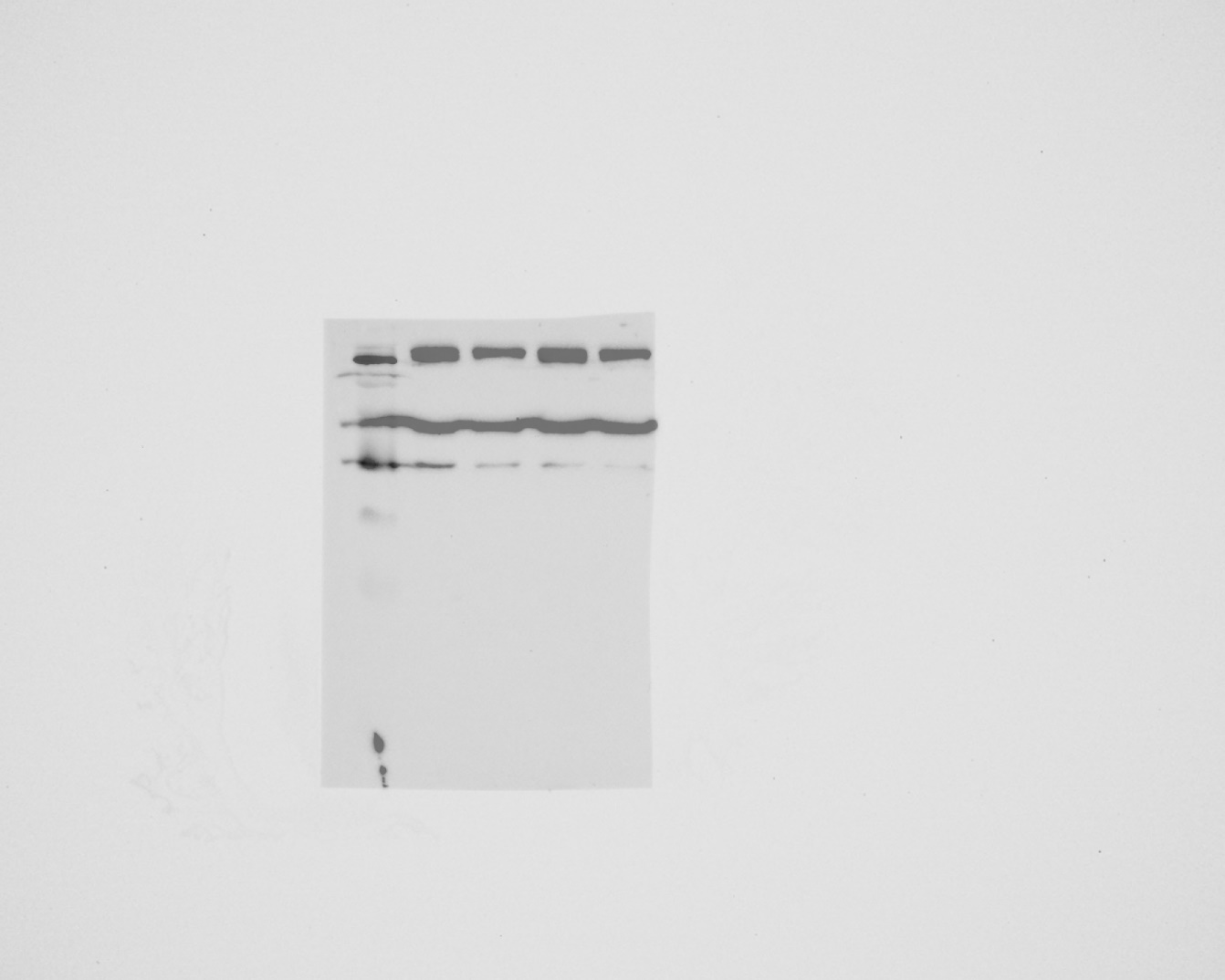

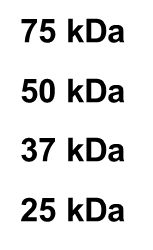

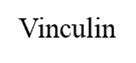


**
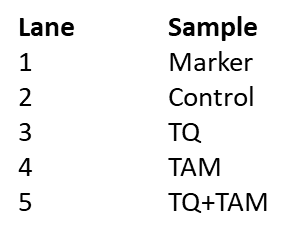
**


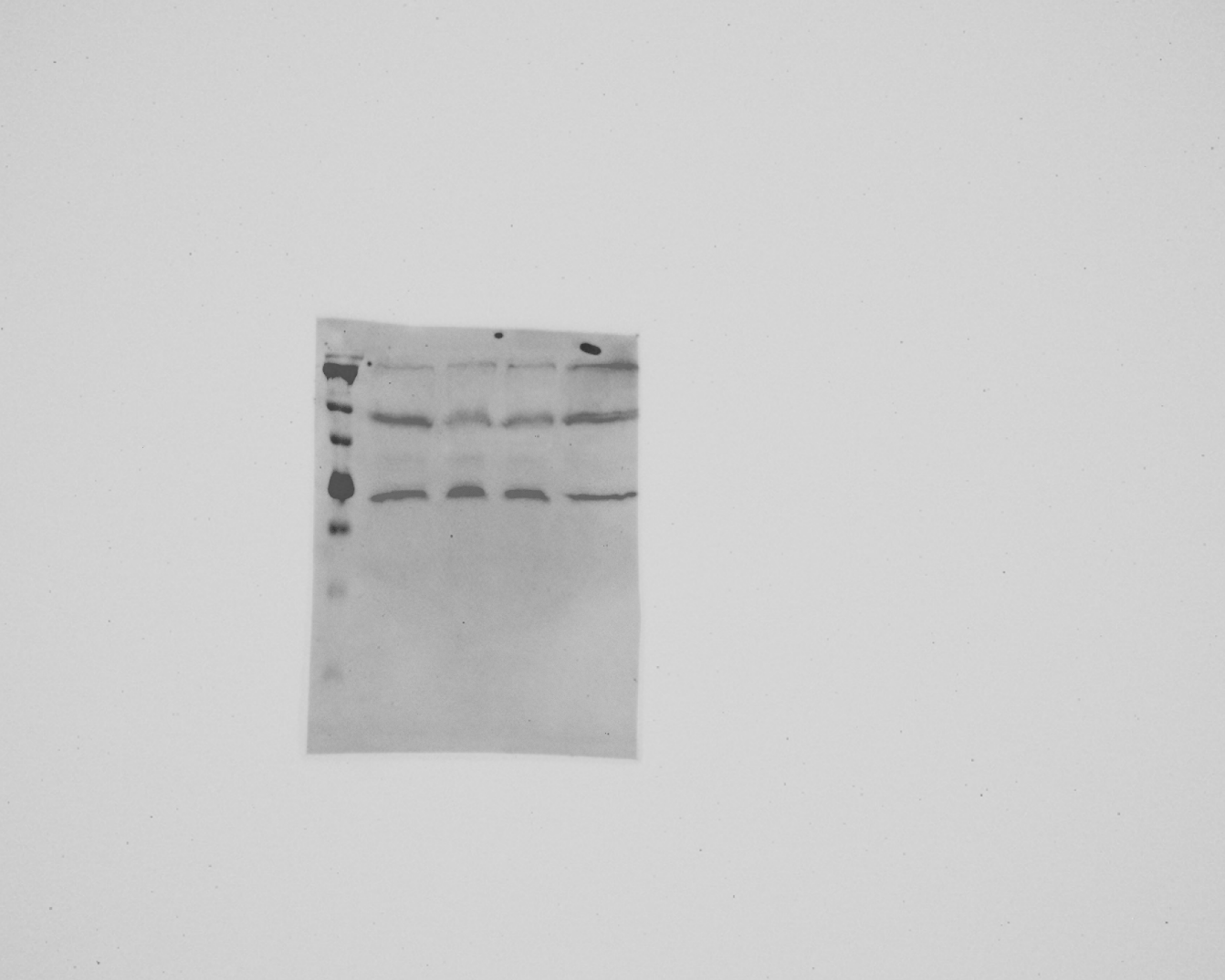


E-cadherin


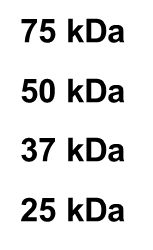

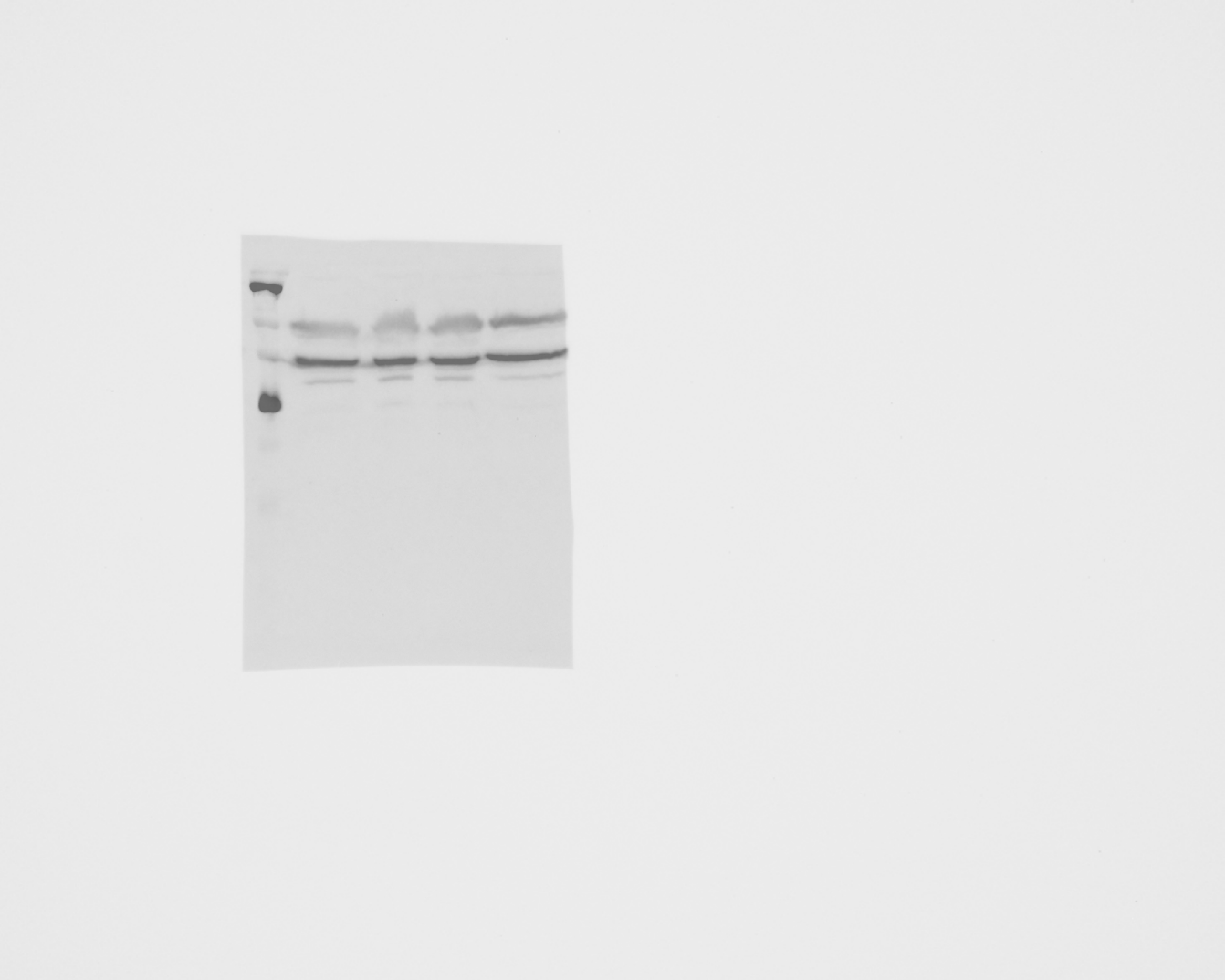


GAPDH


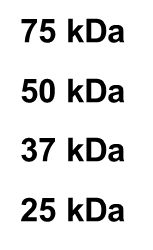


**Densitometry analysis of western blots**

Densitometric quantification of western blot bands was performed using ImageJ 1.54g software (NIH, USA) following a standardized workflow to ensure consistency across all samples and blots. Chemiluminescent blot images were captured, and the original images were used for analysis. Below is the detailed stepwise procedure for ImageJ analysis for densitometry

File-Open-go to the folder-select the WB image-open-navigate to image-type-8-bit-select the rectangular box- take the cursor to the bands and starting from first band drag a box covering the first band and enter 1- further drag the same box to the subsequent band and enter 2- drag again the box to the next bands like wise till the last band and enter 2- Even entering 2 it will show the exact band numbers 2, 3, 4, etc. finally enter 3- another page will appear showing the pixels in the form of curves for each band- pick the straight line from the Image J navigation and make a straight line starting from one end and drag to the other end of the bases of the curve in order to give an area under the curve (only one per band)- next select the wand tool from the Image J and enter in the selected area under the curve region- another page will open and show the results (area)- transfer them in the excel and put in corresponding treatment group (band) for further normalization and fold change calculations.

**Normalization**

The integrated density value of each target protein band was normalized to the integrated density of its corresponding GAPDH band from the same lane to correct for loading variations.

Normalized intensity = Target band intensity / GAPDH band intensity

**Fold Change**

Control = Normalized control / Normalized control

TQ = Normalized TQ / Normalized control

TAM = Normalized TAM / Normalized control

TQ+TAM = Normalized TQ+TAM / Normalized control

**Supplementary Table SI:** List of primers used to amplify target gene using q-PCR. Table represents EMT associated genes primer sequence (Forward and reverse) and their Tm along with amplicon size.

| **Target gene** | **Primer** | **Tm** | **Amplicon size**  **(bp)** |
| --- | --- | --- | --- |
| VIMENTIN Forward | 5’ GGACCAGCTAACCAACGACA 3’ | 62.4 | 178 |
| VIMENTIN Reverse | 5’ AAGGTCAAGACGTGCCAGAG 3’ | 62.4 |  |
| CDH2 Forward | 5’ TGCGGTACAGTGTAACTGGG 3’ | 60.4 | 123 |
| CDH2 Reverse | 5’ GAAACCGGGCTATCTGCTCG 3’ | 62.4 |  |
| SNAI1 Forward | 5’ CACTATGCCGCGCTCTTTC 3’ | 62.4 | 113 |
| SNAI1 Reverse | 5’ GGTCGTAGGGCTGCTGGAA 3’ | 62.4 |  |
| ZEB1 Forward | 5’ AACTGCTGGGAGGATGACAC 3’ | 60.0 | 75 |
| ZEB1 Reverse | 5’ TCCTGCTTCATCTGCCTGA 3’ | 61.0 |  |
| GAPDH Forward | 5’ TGCACCACCAACTGCTTAGC 3’ | 60.4 | 87 |
| GAPDH Reverse | 5’ GGCATGGACTGTGGTCATGAG 3’ | 62.4 |  |
